# Autophagy and neuroprotection in astrocytes exposed to 6-hydroxydopamine is negatively regulated by NQO2: relevance to Parkinson’s disease

**DOI:** 10.1038/s41598-023-44666-7

**Published:** 2023-12-07

**Authors:** Elzbieta Janda, Maddalena Parafati, Concetta Martino, Francesco Crupi, Jonahunnatha Nesson George William, Karine Reybier, Mariamena Arbitrio, Vincenzo Mollace, Jean A. Boutin

**Affiliations:** 1 Laboratory of Cellular and Molecular Toxicology, Department of Health Science, University “Magna Græcia” of Catanzaro, 88100 Catanzaro, Italy; 2 Center for Advanced Studies and Technology (CAST), “G. d’Annunzio” University of Chieti-Pescara, 66100 Chieti, Italy; 3 UMR 152 Pharma-Dev, Université de Toulouse III, IRD, UPS, 31400 Toulouse, France; 4 Institute for Biomedical Research and Innovation (IRIB), National Research Council of Italy (CNR), 88100 Catanzaro, Italy; 5 Laboratory of Neuroendocrine Endocrine and Germinal Differentiation and Communication (NorDiC), Univ Rouen Normandie, Inserm, NorDiC UMR 1239, 76000 Rouen, France; 6 Present Address: Department of Pharmacodynamics, University of Florida, Gainesville, FL 32611 USA

**Keywords:** Biochemistry, Cell biology, Drug discovery, Molecular biology, Diseases, Neurology, Pathogenesis

## Abstract

Dopaminergic degeneration is a central feature of Parkinson’s disease (PD), but glial dysfunction may accelerate or trigger neuronal death. In fact, astrocytes play a key role in the maintenance of the blood–brain barrier and detoxification. 6-hydroxydopamine (6OHDA) is used to induce PD in rodent models due to its specific toxicity to dopaminergic neurons, but its effect on astrocytes has been poorly investigated. Here, we show that 6OHDA dose-dependently impairs autophagy in human U373 cells and primary murine astrocytes in the absence of cell death. LC3II downregulation was observed 6 to 48 h after treatment. Interestingly, 6OHDA enhanced NRH:quinone oxidoreductase 2 (NQO2) expression and activity in U373 cells, even if 6OHDA turned out not to be its substrate. Autophagic flux was restored by inhibition of NQO2 with S29434, which correlated with a partial reduction in oxidative stress in response to 6OHDA in human and murine astrocytes. NQO2 inhibition also increased the neuroprotective capability of U373 cells, since S29434 protected dopaminergic SHSY5Y cells from 6OHDA-induced cell death when cocultured with astrocytes. The toxic effects of 6OHDA on autophagy were attenuated by silencing NQO2 in human cells and primary astrocytes from *NQO2*−/− mice. Finally, the analysis of Gene Expression Omnibus datasets showed elevated NQO2 gene expression in the blood cells of early-stage PD patients. These data support a toxifying function of NQO2 in dopaminergic degeneration via negative regulation of autophagy and neuroprotection in astrocytes, suggesting a potential pharmacological target in PD.

## Introduction

PD is a neurodegenerative disorder mainly characterized by progressive loss of dopaminergic neurons in the *substantia nigra*. The exact mechanisms of dopaminergic neuronal loss in the SN are not fully understood, but several factors have been implicated in the onset and progression of PD. Among them are mitochondrial damage and oxidative stress (OS) caused by alterations in iron and redox metabolism and/or excessive production of toxic dopamine metabolites, followed by protein misfolding and aggregation associated with defective autophagy and “prion-like protein infection”^1–4^.

Autophagy is a catabolic process responsible for the removal of protein aggregates and damaged organelles via lysosomal digestion^5,6^. Autophagy machinery is also involved in the regulation of related processes with an increasingly recognized role in PD, such as vesicular transport, exocytosis and phagocytosis^5,7^. Numerous genetic mutations encoding components of the autophagic machinery have been identified as disease risk factors^1,8,9^. Notably, autophagy dysregulation is also associated with reactive oxygen species (ROS) in both cellular signaling and damage^10^. In particular, all chemical agents inducing ROS and dopaminergic damage, known as Parkinsonian toxins, have been shown to dysregulate or impair autophagy^1,11^ in both neurons and astrocytes.

Astrocytes maintain redox balance in the brain through antioxidant production and detoxification^12^, contribute to the formation and maintenance of the blood–brain barrier^13^ and regulate inflammatory responses in the central nervous system^14,15^. Increasing evidence highlights the contribution of astrocyte dysfunction to the pathogenesis of several neurodegenerative conditions, including PD^16^. DA metabolism is the main source of ROS in the brain^17^. Functional glial cells protect neurons against OS by metabolizing DA via monoamine oxidase-B (MAO-B) and catechol-*O*-methyltransferase (COMT) and by a battery of antioxidant enzymes present in astrocytes^18^. Prolonged dysfunction of astrocytes, involving autophagy and ROS detoxification processes, could increase the vulnerability of DA neurons and advance their degeneration during aging, leading to PD^16,19^.

6OHDA is widely used to investigate the pathogenesis of PD in preclinical studies since it induces PD-like symptoms in rodents^20^. 6OHDA is a DA analog that can be produced by DA hydroxylation in the presence of Fe^2+^ iron and H_2_O_2_. 6OHDA is considered to be a purely synthetic toxin, but several observations suggest the possibility of its endogenous origin, likely during DA catabolism. Indeed, it has been detected in caudate samples as well as in the urine of PD patients^21,22^ and in mouse brains following long-term L-dopa administration^23^. It shows specific PD-like effects^24,25^ due to its toxic oxidation metabolites. Upon injection in the brain, it causes selective death of dopaminergic neurons and proinflammatory activation of microglia, while its effects on the astrocytic compartment are a matter of debate^26,27^. The mechanisms of 6OHDA toxicity are complex and involve rapid auto-oxidation, leading to the generation of hydrogen peroxide and hydroxyl radicals^28^ and impairment of mitochondrial energy production^29,30^. It is also unclear whether any endogenous toxifying enzymes, such as quinone oxidoreductase 2 (NQO2/QR2), may contribute to this process^31,32^.

NQO2 is a cytosolic and ubiquitously expressed flavoprotein that catalyzes the two-electron reduction of quinone to hydroquinone^33^. NQO2, structurally related to NQO1, is considered a phase II detoxifying enzyme, but it enhances the production of activated quinones and ROS in the presence of certain substrates^33,34^. NQO2 emerged as a possible target in PD more than two decades ago. A case–control Japanese study identified a positive association of a nonfamiliar form of PD with a D (deletion) polymorphism in the NQO2 promoter region. This “gain of function” variant of the promoter lacking the Sp3 transcriptional suppressor binding site was 3.46 times more frequent in PD patients than in healthy subjects^35^. Several other data support a potential role of NQO2 in PD and other neurodegenerative diseases^36^. NQO2 may play a role in dopamine metabolism as a catechol quinone reductase^37^ and may negatively influence memory formation and learning processes in mice^38^. Next, chronic OS induced by the parkinsonian toxin paraquat (PQ) is potently inhibited by the specific NQO2 inhibitor S29434/NMDPEF ^39^ in vitro and in vivo in rats following intranigral and systemic injection of PQ^32^. PQ has been shown to inhibit basal autophagy, while treatment with S29434 stimulated autophagy in astrocytes^31^. Recent data indicate that the function of this enzyme is not limited to the generation of OS and inhibition of autophagy, but NQO2 plays a role in flavone-induced autophagy by acting as a receptor of proautophagic ligands, including flavonoids and S29434^40^.

Here, we investigated the toxic effects of 6OHDA and the related role of NQO2 in human and murine astrocytes. We show that 6OHDA potently and dose-dependently inhibited autophagic flux in U373 and primary mouse astrocytes, but S29434 restored and enhanced autophagy in astrocytes and reduced neuronal damage in DA neurons cocultured with astrocytes. A possible role of NQO2 in PD pathogenesis is supported by the higher expression of NQO2 in the majority of PD patients when compared to healthy subjects. These data indicate a toxifying function of NQO2 in dopaminergic degeneration via negative regulation of autophagy and neuroprotection in astrocytes, suggesting it as a potential pharmacological target in PD.

## Results

### Dose-dependent downregulation of autophagy by 6OHDA and stimulation of autophagy by NQO2 inhibition

**Figure 1:**
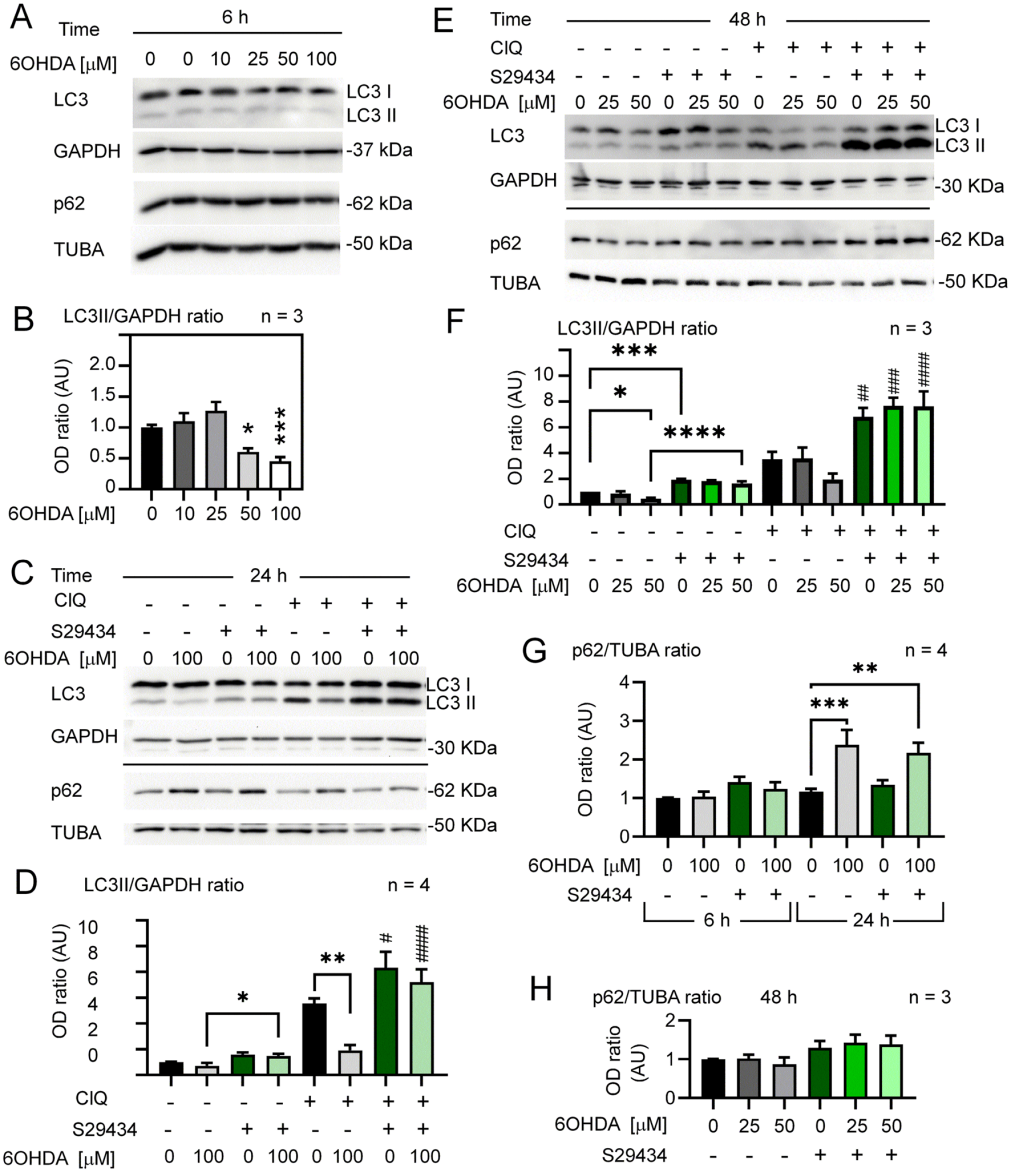
Time- and dose-dependent effect of 6OHDA on autophagic flux. (**A**) Subconfluent U373 astrocytes were exposed to different concentrations of 6OHDA for 6 h before lysis. LC3II and p62 levels were analyzed on separate 15% and 8% SDS-PAGE gels, respectively; GAPDH and α-tubulin (TUBA) were used as loading controls. (**B**) Densitometric (OD) analysis of 3 independent experiments described in (**A**). (**C**) LC3II levels were analyzed in U373 astrocytes exposed to 100 µM 6OHDA +/− S29434 (10 µM) for 24 h. ClQ (25 µM) was added for 2 h before lysis, and protein lysates were analyzed for LC3 and p62 levels on separate gels as described in (**A**). (**D**) OD analysis of 4 independent experiments described in (**C**). (**E**) Cells were treated with 25 or 50 µM 6OHDA +/− S29434 (10 µM) for 48 h. ClQ (25 µM) was added for 3 h before lysis. LC3 and p62 were analyzed by separate 12% and 8% SDS-PAGE, respectively. (**F**) OD analysis of the LC3II TO GAPDH OD ratio in 3 independent experiments described in (**E**). (**G**) OD analysis of the p62 to TUBA ratio after treatment with 6OHDA +/− S29434 (6 and 24 h) in 4 independent experiments described in (**A**) and (**C**). (**H**) OD analysis of the p62 to TUBA ratio after 48 h of treatment with 6OHDA +/− S29434 (10 µM) in 3 independent experiments described in E. The graphs in (**B**), (**D**) and (**F**)–(**H**) show the mean OD ratio +/− SEM, normalized to vehicle-treated controls (0) and expressed in arbitrary units (AU). Statistical analysis: One-way ANOVA followed by Sidak’s post-test in (**B**), (**D**), (**G**) and (**H**) or Welch’s ANOVA followed and unpaired T test with Welch’s correction in (**F**), *p < 0.05, **p < 0.01,***p < 0.001, ****p < 0.0001, when compared to control vehicle-treated cells or as indicated in graphs; #p < 0.05, ^##^p < 0.01, ^###^p < 0.001, ^####^p < 0.001 when compared to the respective 6OHDA-treated and DMSO-treated, S29434-untreated cells.

To examine the effects of 6OHDA on autophagy in astrocytes, we assessed LC3II levels in U373 cells cultured under optimal growth conditions (70–80% confluency, 4.5 g/L glucose). LC3 II levels were not significantly modulated at lower 6OHDA concentrations and were significantly reduced by 50 and 100 µM toxin 6 h after the treatment (Fig. 1A,B) as well as at 24 h (Fig. 1C,D). As expected, we observed an increased reduction in LC3II at 24 h with respect to 6 h (Supplementary Fig. S1).

To assess whether the reduction in LC3II in response to 6OHDA was a true and long-lasting inhibition of autophagic flux, we examined the LC3II levels in the presence and absence of the lysosomal inhibitor chloroquine (ClQ) at 24 h after treatment. In the same experiment, U373 cells were cotreated with the NQO2 inhibitor S29434 to address whether LC3II modulation by 6OHDA was NQO2-dependent. Indeed, 6OHDA (100 mM) had a strong negative effect on autophagic flux at 24 h (Fig. 1C,D), as well as 50 mM at 48 h (Fig. 1E,F). Treatment with 25 mM 6OHDA had no significant effect on LC3II induction (Fig. 1E,F). The control samples in the presence of ClQ were several times higher but reduced by more than 50% or unchanged in 6OHDA-treated samples, depending on the dose, thus confirming a suppression of LC3II by higher concentrations of this toxin. Remarkably, the addition of S29434 potently restored LC3II levels in 6OHDA-intoxicated U373 cells compared to cells without S29434 (Fig. 1C–F). This was associated with a significant increase in p62/SQSTM1 levels after 24 h (Fig. 1C,G) but not at 6 and 48 h posttreatment (Fig. 1A,E,G). Interestingly, a similar time-dependent pattern of induction was observed for NQO2 (Supplementary Fig. S2).

### The effects of 6OHDA on ROS levels

**Figure 2:**
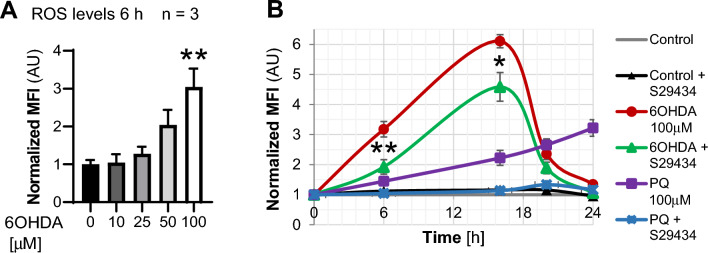
The effects of 6OHDA and NQO2 inhibition by S29434 on oxidative stress. (**A**) 6OHDA-induced oxidative stress was assessed 6 h after treatment by DCFH-DA staining and fluorescence measurement in live U373 cell cultures. The graph shows the mean fluorescence intensity (MFI) +/− SEM from 3 independent experiments normalized to the MFI of control vehicle-treated cells. Statistical analysis: One-way ANOVA followed by Sidak’s posttest, **p < 0.01, compared to control vehicle-treated cells. (**B**) Inhibition of oxidative stress by S29434. U373 cells were treated for 6, 16, 20 and 24 h with 100 µM 6OHDA or 100 µM PQ +/− S29434 (10 µM), incubated with MitoSox, detached by trypsin and analyzed by flow cytometry. The graph shows the mean fluorescence intensity (MFI) +/− SEM, normalized to untreated controls in 3 independent experiments performed at least in duplicate. Statistical analysis: unpaired T test, *p < 0.05, **p < 0.01 compared to the respective 6OHDA-treated, DMSO-treated and S29434-untreated cells.

Astrocytes are known for their capacity to counteract OS induced by various sources. Therefore, the lack of autophagy stimulation observed here could depend on the absence of OS in response to 6OHDA. For this reason, we measured the OS induced by 6OHDA. Interestingly, the fluorescein derivative DCF-DA detected a significant OS increase only at higher doses of 6OHDA (Fig. 2A), which effectively suppressed autophagy (Fig. 1B), suggesting that 6OHDA-induced OS inhibits autophagy, similar to what was demonstrated for PQ-induced OS^31^. Next, we compared PQ- to 6OHDA-induced OS and the impact of S29434 on ROS formation in response to both compounds, as detected by the fluorescent probe MitoSox. This dye monitors the temporal changes in hydroxyradical and superoxide anions in the mitochondria and cytoplasm. 6OHDA (100 µM) induced a time-dependent increase in MitoSox fluorescence, with a remarkable peak of activity 15 h posttreatment (Fig. 2B), much stronger than in the case of 100 µM PQ. Cotreatment with S29434 (10 µM) attenuated ROS induction by 6OHDA at each tested time point, but the effect was only partial, ranging from a 30% to 50% reduction, compared to the almost full attenuation of the MitoSox signal in the presence of PQ and S29434 (Fig. 2B). Thus, OS induced by 6OHDA is more potent but less persistent and less sensitive to S29434 than PQ-induced OS. Nevertheless, the findings in Figs. 1 and 2 indicate that 6OHDA-induced toxicity suppresses basal autophagy, which can be equally well restored by S29434 in astrocytes as previously shown for PQ, despite important differences in OS dynamics between both parkinsonian toxins.

### 6OHDA effects on cellular and in vitro NQO2 activity

**Figure 3:**
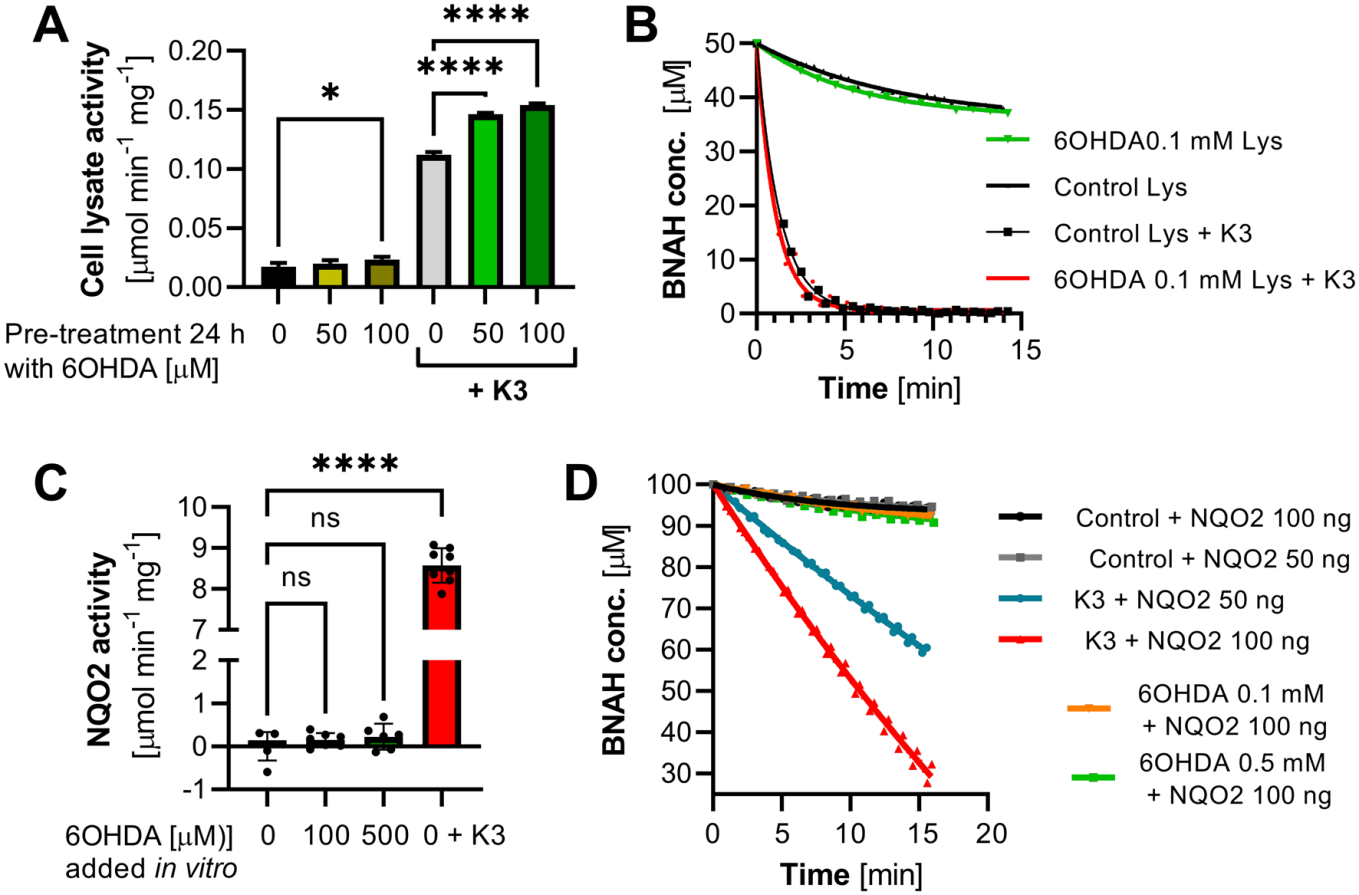
6OHDA stimulates NQO2 activity in astrocytes, although 6OHDA is not an NQO2 substrate in vitro. (**A**) NQO2 activity was measured in lysates (Lys) of U373 cells exposed to 6OHDA (50 or 100 mM) for 24 h before cell lysis. The reactions were carried out with BNAH (50 mM) as a cosubstrate and 9–14 mg of cell lysates in the presence or absence of K3 (50 mM) as a substrate. The graph shows the mean +/− SD of one representative experiment performed in triplicate out of 4 independent experiments that yielded similar results. (**B**) Representative BNAH decay curves of the experiment described in A. (**C**) Testing 6OHDA as a substrate for NQO2 in an activity assay with recombinant human NQO2 (50 or 100 ng/200 µL) and 0.1 mM BNAH as a cosubstrate. K3 (100 mM) was used as a positive control. The velocity of BNAH decay was calculated from the first 5 min of the reactions presented in D and normalized to control reactions (BNAH + enzyme/no substrate). The graph shows the mean +/− SD of two representative experiments performed at least in triplicate. (**D**) Representative BNAH decay curves of the experiment described in (**C**). Statistical analysis in (**B**) and (**C**): One-way ANOVA followed by Sidak’s posttest; *p < 0.05, ****p < 0.0001 or ns (not significant) when compared to control bars as indicated.

Next, we addressed whether 6OHDA can influence cellular NQO2 activity in astroglial cells when added to cell culture. To this end, U373 cells were treated for 24 h with 6OHDA (50 and 100 mM), and cell lysates were assayed for their capacity to reduce menadione (K3) and oxidase BNAH, which are commonly used substrates and cosubstrates, respectively, for the detection of NQO2 activity. U373 cell lysates pretreated with 6OHDA showed higher NQO2 activity than control cells in the presence of K3 but also when no exogenous substrate was used (Fig. 3A,B). Next, we addressed whether 6OHDA or its autooxidation products, which are rapidly produced in solution^41^, might be substrates of the quinone oxidoreductase activity in vitro. 6OHDA was added as the only substrate in the reaction mixture containing purified recombinant human NQO2 and BNAH as a cosubstrate. 6OHDA dose-dependently tended to accelerate BNAH oxidation, but this effect was not statistically significant when compared to spontaneous BNAH decay in the presence of the enzyme (without substrate) and more than 50 times lower when compared to NQO2 activity in the presence of K3 (100 mM), which was used as a positive control (Fig. 3C,D). These data indicate that 6OHDA is not a substrate of NQO2, but 6OHDA dose-dependently induces NQO2 activity in astrocytes, which can be well explained by an increased expression of NQO2 in response to 6OHDA (Supplementary Fig. S2).

### U373 astrocytes are resistant to 6OHDA-induced toxicity in optimal culture conditions in dopaminergic neuroblastoma cells

**Figure 4:**
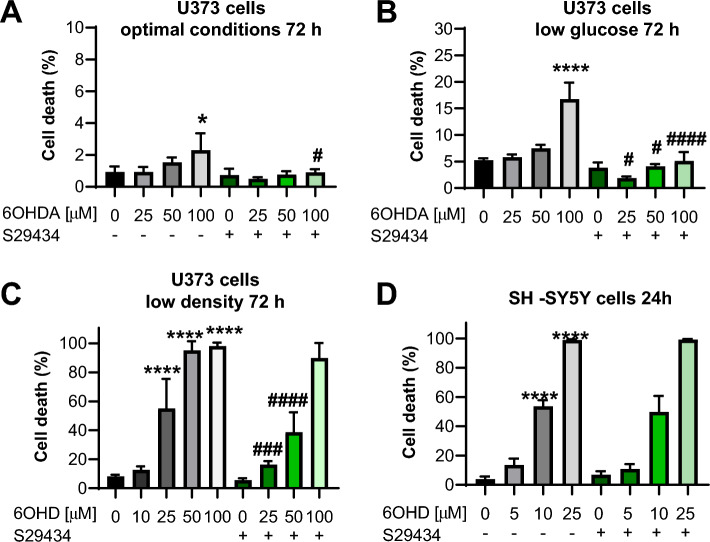
Specific NQO2 inhibitor S29434 reduces 6OHDA-induced mortality of U373 cells in stressful culture conditions but has no effect on viability of dopaminergic neuroblastoma cells. (**A**) U373 astrocytes were cultured in regular growth conditions, exposed to 25–100 µM 6OHDA for 72 h −/+ S29434 (10 µM) and then harvested by trypsin-mediated detachment. (**B**) U373 cells were cultured in low glucose DMEM for 2 days and then exposed to 25–100 µM 6OHDA for 72 h −/+ S29434 (10 µM). (**C**) U373 cells were plated at 5 times lower density than usual conditions. After 2 days, they were exposed to 10–100 µM 6OHDA for 72 h −/+ S29434 (10 µM). (**D**) SH-SY5Y cells were plated in DMEM-high glucose with RPMI 1640 and exposed to 5–25 µM 6-OHDA for 24 h −/+ NMDPEF (10 µM). (**A**–**D**) Cell mortality was analyzed by trypan blue exclusion assay and flow cytometry (TBF). The graphs show the mean +/− SD of a representative experiment out of three independent experiments performed in triplicate. Statistical analysis: One-way ANOVA followed by Sidak’s posttest; *p < 0.05, ****p < 0.0001 when compared to control vehicle-treated cells; ^#^p < 0.05, ^###^p < 0.001, ^####^p < 0.0001 when compared to respective DMSO-treated, S29434 untreated cells.

The negative effect on autophagy in U373 astroglial cells might be a consequence of general 6OHDA-induced toxicity leading to cell death. Nevertheless, we were able to detect only background cell death (≈2%) in optimal culture conditions, which were standard conditions applied in all autophagy experiments (Fig. 4A). To detect significant cell mortality in response to 6OHDA, U373 cells had to be exposed to additional stress factors, such as incubation in low glucose after high-glucose medium (Fig. 4B) or seeding at very low density (Fig. 4C). Interestingly, S29434 efficiently reduced cell death, also in suboptimal growth conditions, partially dependent on the presence of the toxin (Fig. 4B,C), with a significant effect at lower concentrations of 6OHDA (25 and 50 µM) in sparsely seeded U373 cells (Fig. 4C).

Importantly, U373 cells overexpressing NQO2, were more sensitive to 6OHDA and showed higher mortality than U373 cells when assessed at a low cell density (Supplementary Fig. S3), thus supporting a toxifying effect of NQO2 in cells exposed to 6OHDA. Next, we examined the potential involvement of NQO2 in 6OHDA–induced toxicity in neuroblastoma cells (SH-SY5Y), a commonly used in vitro model for dopaminergic neurons. SH-SY5Y cells were much more sensitive to 6OHDA than U373 cells and were massively dying 24 h after the addition of 10 µM 6OHDA. Treatment with S29434 did not prevent cell death in SH-SY5Y cells (Fig. 4D).

Thus, 6OHDA reduces autophagic flux in U373 cells, even though these cells are highly resistant to 6OHDA-induced cell death. This contrasts with SH-SY5Y cells that rapidly die in response to 6OHDA and are not protected by S29434.

### Suppression of basal autophagy by 6OHDA in U373 cells and primary murine astrocytes is dependent on NQO2 status

**Figure 5:**
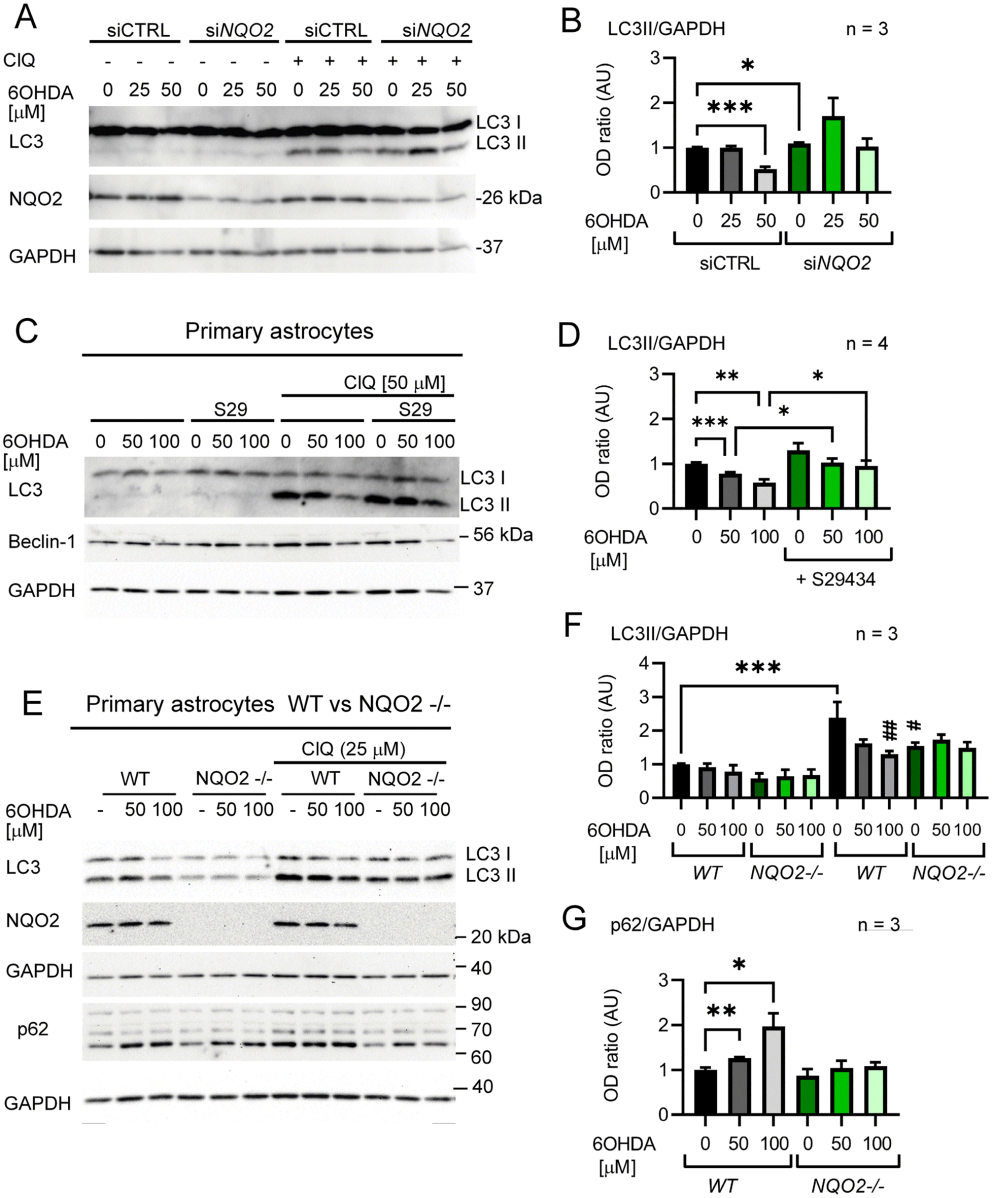
(**A**), (**B**) Silencing of NQO2 restores autophagy in U373 cells exposed to 6OHDA. (**A**) U373 cells were transfected with no-target siRNA (siCTRL) or siRNA targeting human *NQO2* (si*NQO2*). Twenty-four hours after the end of transfection, the cells were exposed to 6OHDA (25–50 µM) for 24 h. Three hours before lysis, the cells were treated with ClQ (25 µM). LC3 expression was analyzed by 12% SDS-PAGE followed by WB. B) Analysis of the LC3II to GAPDH OD ratio in 3 independent experiments described in A. Only LC3II in the presence of ClQ was analyzed. Statistical analysis: Welch’s ANOVA test followed by unpaired t test with Welch’s correction. (**C**), (**D**) The toxifying effects of 6OHDA and NQO2 on autophagy in mouse primary cortical astrocytes. (**C**) A representative blot of LC3, Beclin-1 and GAPDH expression in primary cortical astrocytes treated with 6OHDA (50 and 100 µM) +/− S29434 (10 µM) for 24 h. The treatments were performed on dense, 95% confluent cultures. (**D**) Analysis of the LC3II to GAPDH OD ratio in 3 independent experiments described in (**C**). Statistical analysis: Welch’s ANOVA followed by unpaired t test with Welch’s correction. (**E**), (**F**), (**G**) Primary cortical astrocytes from *NQO2* − / − mice are more resistant to 6OHDA-induced autophagic toxicity than their *WT* counterparts. (**E**) A representative blot of LC3, NQO2, p62 and GAPDH expression in both *NQO2* − / − and *WT* primary cortical astrocytes treated with 6OHDA (50 and 100 µM) for 24 h plated as in C, but a different antibody was used for WB as described in the Methods section. (**F**) Analysis of the LC3II to GAPDH OD ratio in 3 independent experiments described in (**E**). Statistical analysis: One-way ANOVA followed by Sidak’s posttest. (**G**) Analysis of the p62 to GAPDH OD ratio in 3 independent experiments described in (**E**). Only p62 in the absence of ClQ was analyzed. Statistical analysis: Welch’s ANOVA test followed by unpaired t-test with Welch’s correction. (**B**), (**D**), (**F**), (**G**) Bars show the mean +/− SEM. *p < 0.05, **p < 0.01, ***p < 0.001 for comparisons as indicated in the graphs; ^#^p < 0.05, ^##^p < 0.01 when compared to control ClQ-treated cells.

Next, we determined whether NQO2 is a key player in 6OHDA-induced autophagy dysfunction. To answer this question, we silenced NQO2 in 6OHDA-treated U373 cells. Our results indicated a dose-dependent decrease in LC3II levels in cells 24 h after treatment with 25–50 µM 6OHDA (Fig. 5A,B). However, *NQO2* silencing restored or enhanced autophagy in cells exposed to higher (50 µM) and lower (25 µM) doses of toxin, respectively, although the effect was statistically significant only with 50 µM 6OHDA (Fig. 5B). In fact, a stimulatory effect on LC3II levels was statistically significant in cells expressing lower levels of NQO2 (si*NQO2*) in response to 25 µM 6OHDA doses when analyzed by less stringent statistical tests (unpaired T test, P = 0.0418; one-way ANOVA and uncorrected Fisher’s LSD test, P = 0.0145). In conclusion, the silencing of NQO2 partially restores autophagy in cells exposed to 6OHDA. These data support the importance of NQO2 in toxin-induced autophagy defects in human astrocytes.

To confirm the glioprotective effect of S29434 in astrocytes, we established primary cultures of cortical astrocytes from C3H mice and treated them with 6OHDA (50 and 100 µM) in the absence and presence of S29434. These concentrations of the parkinsonian toxin were not able to induce any significant cell death (data not shown), but WB analysis revealed that 24 h treatment with 6OHDA inhibited autophagic flux by decreasing LC3II levels in a dose-dependent fashion. This was evident only in the presence of ClQ, since in these experimental conditions (90–100% confluent monolayers), it was more difficult to detect LC3II without preincubation with ClQ (Fig. 5C). Of note, S29434 increased LC3II levels in cells exposed to 6OHDA, indicating the involvement of NQO2 in the negative regulation of autophagy in murine astrocytes under 6OHDA stress, although it did not induce a statistically significant increase in LC3II levels in control cells (Fig. 5D). To further assess the role of NQO2 in autophagic flux regulation upon 6OHDA intoxication, we analyzed LC3II levels in primary cortical astrocytes from mice lacking NQO2 (*NQO2-/-*) and compared them with their *wild-type* (*WT*) counterparts. These cells were plated and analyzed under the standard protocol, but another anti-LC3 antibody was employed, allowing LC3II detection w/o ClQ. The analysis of LC3 expression upon 24 h of treatment with 6OHDA revealed a downregulation of LC3II only in *WT* but not in KO astrocytes (Fig. 5E), and the difference was statistically significant in the presence of ClQ (Fig. 5F). The absence of NQO2 also attenuated the upregulation of p62 in response to 6OHDA (Fig. 5E,G), which suggests a role of NQO2 in the regulation of autophagy in astrocytes in response to oxidative insults.

### S29434 enhances the protective effect of U373 cells on dopaminergic SH-SY5Y cells against 6OHDA-induced cell death in coculture experiments

**Figure 6:**
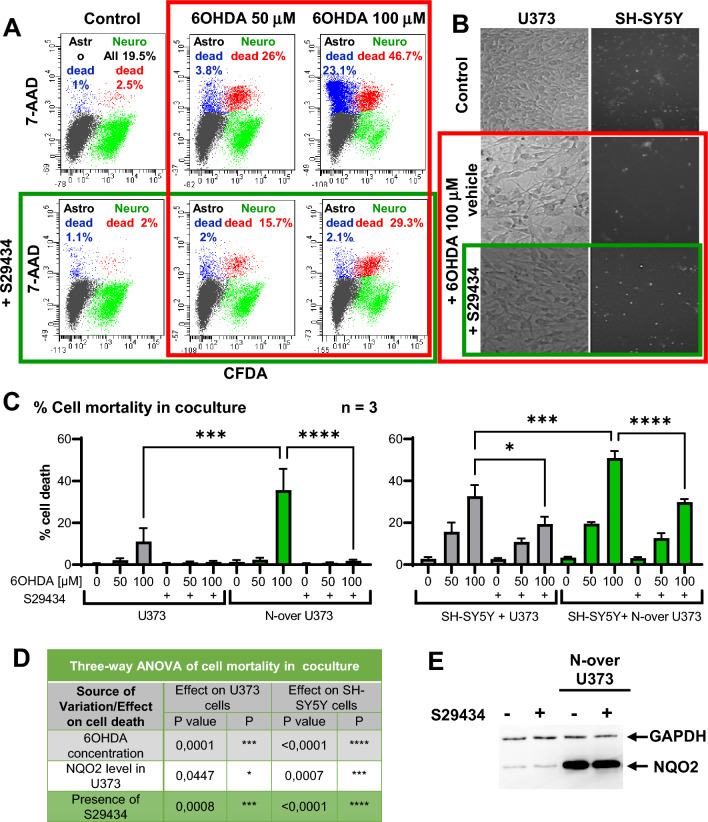
Dopaminergic SH-SY5Y cells are protected from 6OHDA-induced toxicity in NQO2-dependent manner in coculture experiments with U373 astrocytes. Empty vector- and NQO2-overexpressing (N-over) U373 cells were cultured with SH-SY5Y cells labeled with CMFDA (green). After 24 h of coculture, cells were exposed to 6OHDA +/− 10 µM S29434 for the next 48 h. Cell mortality was analyzed by flow cytometry after 7-AAD staining. (**A**) Representative dot-plots of N-over U373 cells in coculture with SH-SY5Y showing live and dead cell populations (green and red for SHSY5Y cells and black and blue U373 cells, respectively) in a typical experiment as described in A. (**B**) Representative phase-contrast and fluorescence microscope images of cocultures 24 h after treatment with 6OHDA +/− 10 µM S29434 as indicated (magnification 20X). (**C**) Analysis of cell death in 3 independent experiments as shown in A. The left and right graphs show dead U373 and SH-SY5Y cells, respectively (the mean % +/− SEM). Statistical analysis: One-way ANOVA followed by Sidak’s posttest; *p < 0.05, **p < 0.01, ***p < 0.001, ****p < 0.0001. (**D**) The data presented in (**C**) were subsequently reanalyzed by three-way ANOVA (U373 and SH-SY5Y were analyzed separately) to evaluate the effect of 3 experimental variables on cell death. (**E**) WB analysis of NQO2 expression in U373 cells used for the above experiments treated for 24 h with S29434 (10 µM).

The primary role of astrocytes is to protect neurons from oxidative insults. Their neuroprotective capacity can be assessed in vitro in human astrocyte-neuron cocultures. We developed a simple coculture assay by overlaying U373 astrocyte monolayers with SH-SY5Y cells and scoring the viability of the respective cell populations in the presence of toxic insults^31^. In this assay, SH-SY5Y cells were labeled with a green-fluorescent dye (DFCA) before mixing the two cell types. Subsequently, the monolayers of naïve U373 or U373 cells overexpressing NQO2 (N-over cells, Fig. 6E) were overlaid with a DFCA-labeled dopaminergic cells. After 24 h, cocultured cells were exposed to 6OHDA for the next 48 h in the presence or absence of 10 µM S29434 (Fig. 6B). Cell mortality was assessed by flow cytometry with 7-AAD staining (Fig. 6A). We found that dopaminergic SH-SY5Y cells were potently protected by U373 monolayers from 6OHDA-induced cell death, and we could observe SH-SY5Y mortality only at very high doses of 6OHDA (50 and 100 µM), while N-over U373 cells were more sensitive to the neurotoxin and less protective with respect to SH-SY5Y cells (Fig. 6A,C). Importantly, the protective effect of U373 astrocytes was strongly enhanced by S29434, both in normal cells and in N-over cells (Fig. 6A–C). NQO2 overexpression as well as its inhibition by S29434 in astrocytes exerted a highly significant effect, especially for SH-SY5Y cells, when analyzed by three-way ANOVA (Fig. 6D). As expected, the extent of the protection by S29434, calculated after subtracting basal mortality of SH-SY5Y cells in coculture with normal U373 cells (around 3 to 5%), was dependent on the concentration of 6OHDA and was around 44% at 100 µM, but reached full protection at lower doses, such as 10 µM (Fig. S5A and S5B). These experiments demonstrate that modulating NQO2 activity and protein levels has a strong impact on dopaminergic neuron survival when in coculture with astrocytes, in contrast to monoculture, as shown in Fig. 4D.

### The analysis of NQO2 gene expression in PD patients reveals differences compared to healthy individuals

**Figure 7:**
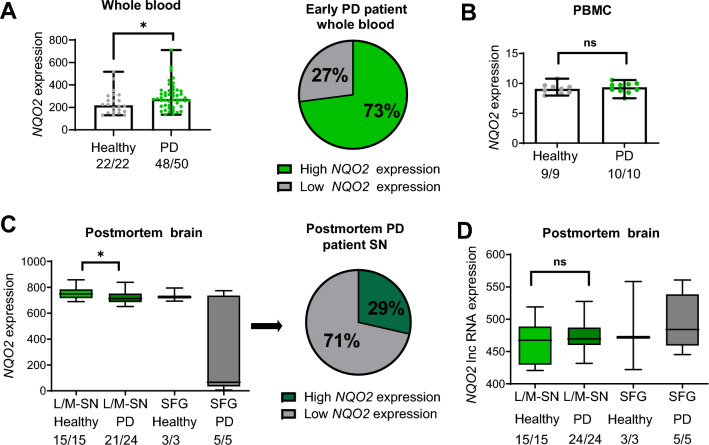
Higher NQO2 gene expression in early-stage PD and its reduction in advanced, postmortem stages of PD. (**A**) The expression of the NQO2 gene (probe set 203814_s) in healthy controls and Parkinson’s disease [PD] patients in whole blood according to GSE6613 datasets. Statistical analysis: Normality tests followed by the Mann–Whitney test. Medians +/− range in the left graph. The pie graph on the right indicates the percentage of PD patients with a higher expression than the median of healthy subjects; (**B**) NQO2 gene expression (probe set TC0600014055.hg.1) in peripheral blood mononuclear cells (PBMCs) from healthy donors and PD patients in GSE100054 datasets. Statistical analysis: Mann–Whitney test. Medians +/− range. (**C**) NQO2 gene expression (probe set 203814_s) in different postmortem brain tissues of healthy and PD donors in the GSE8397 dataset. Statistical analysis: Normality tests followed by unpaired t-tests. Means +/− SD in the left graph. The pie graph on the right indicates the percentage of PD patients with a higher expression than the mean of healthy subjects. (**D**) The expression of the long non-coding RNA (NQO2) gene (probe set 237870_at) in different PD postmortem brain tissues of healthy and PD donors. Statistical analysis: Normality tests followed by unpaired t-tests. SFG: superior frontal gyrus, L/M-SN: lateral/medial substantia nigra. The numbers of analyzed samples on total samples are mentioned below each group. (**A**), (**C**) Very low expression outliers were excluded in the PD groups. *p < 0.05, *ns* not statistically significant.

Thus far, our data suggest that high NQO2 levels or activity may increase the sensitivity of astrocytes and nearby neurons to OS and parkinsonian toxins. However, it is not clear whether high NQO2 levels may predispose patients to PD or other OS-dependent neurodegenerative diseases because *NQO2* gene expression and protein levels have never been analyzed in PD patients. Public gene expression databases are unexplored resources in this respect. We analyzed the Gene Expression Omnibus (GEO) datasets of Affymetrix Human Genome arrays. We identified a few studies that analyzed gene expression in PD patients of thousands of genes, including *NQO2*. One study analyzed genome-wide gene expression in the whole blood of early-stage PD patients (n = 50) and compared them to healthy donors (n = 22)^42^. The analysis of GSE6613 datasets revealed a high variability and nonnormal distribution of *NQO2* expression in early PD patients, but most cases [73%] showed higher expression than the central tendency in healthy patients, which was statistically significant with and without two potential outliers (Fig. 7A). We also observed a higher median *NQO2* expression in white blood cells of PD patients in a smaller dataset (GSE100054) comprising 10 cases and 9 controls, but it was not significant (Fig. 7B)^43^. Finally, we analyzed the GSE8397 datasets from postmortem brain specimens of 24 late-stage PD patients and 16 controls^44,45^ available for two probes targeting the *NQO2* gene (GSE8397-U133A and U133B). Surprisingly, lateral and media *substantia nigra* (L/M-SN) from PD patients presented significantly lower expression of *NQO2* than that from healthy donors, even after excluding three very low expression outliers (p = 0.04, n = 21 vs n = 15). In this case, 71% of PD cases showed lower NQO2 expression compared to the mean of control cases (Fig. 7C). In contrast, the expression levels detected by the second probe, corresponding to long noncoding RNA (lncRNA) of *NQO2* (*lncNQO2*), showed no significant increase in PD samples compared to the mean *lncNQO2* expression levels in healthy samples (Fig. 7D). The GSE8397 dataset also contains the data from the *superior frontal gyrus* (SFG), but owing to the low number of samples (n = 5), it is difficult to draw any conclusions (Fig. 7C,D). These data strongly support a possible involvement of NQO2 in PD etiology.

## Discussion

The important finding of the present work is the identification of NQO2 as a player in autophagy and in the impairment of neuroprotective capacity in astrocytes induced by 6OHDA. This compound is a model dopaminergic neurotoxicant, but may also represent toxic endogenous dopamine metabolites^20,23^. Similar to other parkinsonian toxins, 6OHDA reproduces the main cellular processes of PD , such as OS, neurodegeneration, neuroinflammation, and neuronal death by apoptosis, although it is controversial whether 6OHDA-induced animal PD models accumulate a-synuclein aggregates similar to Lewy bodies, typical features of human PD^46^. The mechanism of 6OHDA toxicity has been linked to ROS production by extracellular auto-oxidation^47^, leading to intracellular OS induction in various cell types^31,48^. In agreement with this, we observed that mitochondrial and cytoplasmic ROS increased in a dose- and time-dependent fashion in U373 cells, but the intensity and duration of the ROS burst were totally different than in the case of PQ. In fact, OS induced by 6OHDA was more rapidly increasing and disappearing and several times stronger than that induced by PQ. Nevertheless, the effects of both neurotoxins on autophagy were similar and consistent with the concept that persistent and/or high levels of OS inhibit rather than stimulate autophagy. Indeed, the exposure of U373 astrocytes to increasing 6OHDA doses had no effect or significantly downregulated autophagic machinery as evidenced by a more than 50% decrease in LC3 lipidation when 100 µM 6OHDA was applied for 24 h. This correlated with a significant increase in p62 levels at 24 h after the addition of 6OHDA (Fig. 1C,G) but not at 48 h (Fig. 1H), as previously found for PQ^31^. It is likely, however, that the lack of p62 upregulation is due to the very low solubility of p62 aggregates that accumulate at later time points. In addition, the exposure of astrocytes to different concentrations of 6OHDA led to no or marginal lethality under optimal culture conditions. In contrast, in our previous study, we were not able to distinguish between PQ-induced general toxicity and PQ-dependent autophagy dysfunction in astrocytes, since a clear reduction in autophagic flux preceded massive cell death that took place at 72 h^31^. Thus, despite evident differences in OS dynamics and cellular toxicity, both toxins efficiently lead to autophagy dysfunction in astrocytes.

In contrast to the data presented here, a net induction of autophagy was widely reported for dopaminergic neurons in vitro in response to 6OHDA, MPTP, and other parkinsonian toxins^11^. However, this apparent autophagy stimulation in vitro by 6OHDA is not sufficient since many potent autophagy inducers protect dopaminergic neurons from cell death^1,49,50^. A net induction of autophagy does not occur in astrocytes, likely because certain inhibitory pathways activated by toxins, comprising the NQO2 pathway reported here, counterbalance any proautophagic signals triggered by OS. This concept is well supported not only by our observations in astrocytes^1,31^ but also by other authors in several alternative models of PD based on neurotoxicants^51–55^. However, robust evidence of net autophagic flux modulation by 6OHDA or other dopamine metabolites in *vivo* is missing. Only one paper that investigated 6OHDA effects on autophagy in rodents reported LC3II and p62 upregulation in lesioned SN samples, which would suggest inhibition of autophagic flux, but these findings were relative to 4 weeks upon treatment and were not confirmed by cotreatment with lysosomotropic agents^50^. Importantly, all authors agree that pharmacological stimulation of autophagy by Torin1, curcumin or NRF2 inducers in 6OHDA-treated rodents correlates with neuroprotective effects^49,50,55^, suggesting that 6OHDA causes defective rather than excessive autophagy.

When autophagic flux is reduced by decreased LC3II production, autophagosome transport blockade and/or defective lysosomal function, specialized forms of autophagy, such as mitophagy are also impaired, leading to the accumulation of dysfunctional mitochondria. Autophagy and mitophagy impairment by genetic or epigenetic mechanisms is strictly linked to the progression of PD^1,9^, and recent reports suggest that a possible autophagy/mitophagy defect may reside in astrocytes^19,56^. With emerging evidence demonstrating a broader role of glial mitochondria in detoxification processes^57^ and astrocyte dysfunction in PD pathogenesis^16,19^, it is likely that parkinsonian toxins, such as 6OHDA and PQ, damage mitochondria and impair glial mitophagy at the same time, leading to the impairment of their neuroprotective capacity, which heavily relies on mitochondrial function in astrocytes^57,58^. Therefore, it is possible that mitochondria are involved in the neuroprotective effect of the NQO2 inhibitor presented in this work (Fig. 6), even though NQO2 is a cytoplasmic enzyme^33^, and preliminary experiments exclude its mitochondrial localization (E. Janda personal communication). Future studies should shed more light on a possible role of NQO2 in mitophagy and mitochondrial function in the glial compartment.

A potential role of NQO2 in the neurodegenerative process is also supported by its upregulation of both protein levels and activity in cells exposed to 6OHDA (Fig. 3; Fig. S2). This observation is in line with our previous findings obtained with cells exposed to PQ and then assayed in reactions containing BNAH and K3^32^. In addition, we showed that cells pretreated with 6OHDA sustained faster BNAH oxidation in the absence of exogenous substrates, such as K3 (Fig. 3A), suggesting that 6OHDA metabolism leads to the production of quinones that act as substrates for NQO2 in vivo. Such quinones are not produced in vitro and 6OHDA itself is not a substrate for NQO2 (Fig. 3C) but could be produced in cells*.* Our previous results obtained with PQ also support this hypothesis^31^, but futher experiments are required to validate it.

Another novel finding of the present work is that NQO2 contributes much less to 6OHDA-induced ROS induction than to autophagy impairment in astrocytes. In fact, S29434 almost doubled autophagy levels in control cells and fully restored or even upregulated autophagic flux in U373 cells exposed to 6OHDA (Fig. 1D,F). In contrast, S29434 had a moderate effect on OS induced by the toxin, reducing it by 25% at the peak oxidative burst (Fig. 2B), suggesting that the main beneficial activity of the compound is linked to autophagy stimulation. However, the murine astrocytes were less responsive to S29434, since the compound produced a significant stimulation of autophagy only in 6OHDA-treated cells (Fig. 5C,D). Nevertheless, the experiments with *NQO2* − / − primary astrocytes provide key evidence for an important role of NQO2 in the negative regulation of LC3II induction. In contrast to *WT* cells, astrocytes lacking NQO2 were resistant to 6OHDA-induced LC3II downregulation, and the upregulation of p62 was significantly reduced in these cells, indicating that autophagic flux was less impaired by 6OHDA in the absence of NQO2. In addition, we observed a statistically significant reduction in LC3II levels and lower p62 expression in knockout cells, indicating differences in autophagic machinery between *WT* and *NQO2*-deficient astrocytes. To our knowledge, this is the first analysis of autophagic marker expression in *NQO2* − / − cells. Overall, these and our previous data as well as a recent work reporting that negative regulation of autophagy by fluoride is NQO2-dependent^59^ strongly suggest that NQO2 is an important modulator of autophagy. The question of whether it is mediated by its enzymatic activity or by the formation of NQO2 complexes with its protein partners hypothetically regulated by S29434 and its other inhibitors, as suggested previously^39^, needs to be further investigated.

Another important issue is the relevance of our in vitro model to complex pathogenic mechanisms in PD. The causative role of toxic dopamine metabolites in PD pathogenesis is well supported by several observations^60^, but robust evidence of autophagic flux inhibition by 6OHDA or other dopamine metabolites in vivo is missing in the literature and in the present paper. In addition, the coculture of U373 cells with SH-SY5Y cells provides a proof of concept that the toxicity of 6OHDA to neurons can be attenuated by modulating NQO2 levels and activity in astrocytes, but it is far from modelling complex interactions that take place *in* vivo. Thus, to address the NQO2 role in PD, it would be necessary to examine first 6OHDA effects on autophagy in vivo and then to verify the protective effects of NQO2 inhibitors. On the other hand, induced pluripotent stem cell-derived organoids of dopaminergic neurons and astrocytes would be a better model to study the role of NQO2 in neuroprotective capacity of astrocytes^61^.

In spite of these weak points, our study provides important clinical evidence supporting a possible role of NQO2 in PD progression is further supported by another important finding presented here, i.e., the difference in expression levels of *NQO2* between PD patients and healthy donors. Higher expression of *NQO2* in PD was first suggested by Harada et al.^35^, who described a positive association of a common form of PD with the D (deletion) polymorphism in the *NQO2* promoter region. This “gain of function” genetic variant of the promoter lacking the Sp3 transcriptional suppressor binding site^62^ was 3.46 times more frequent in PD patients than in healthy subjects in the Japanese population^35^. Only two attempts to reproduce the findings of Harada’s work have been done so far, and one was discordant^63^, while another minor work confirmed them^64^. More recent and extensive studies of *NQO2* gene or protein expression in PD patients are missing, but higher NQO2 protein levels were reported in postmortem brain specimens of patients with Alzheimer’s disease (AD)^65^, and elevated NQO2 expression was convincingly associated with learning deficits in rodent models^38^. The data retrieved from GSE data sets suggest the opposite in postmortem SN specimens of late-stage PD patients, namely, a statistically significant reduction in *NQO2* mRNA levels. This is in line with a higher expression of lnc*NQO2*, which might repress *NQO2* expression^66^. These data are unexpected, but do not exclude the possibility that higher *NQO2* expression might be a trigger or risk factor for neurodegenerative changes in the early stages of PD. This hypothesis is supported by the evidence coming from the analysis of *NQO2* expression in whole blood of 48 early PD cases, which revealed that the majority (35/48 (≈73%) of early PD patients express higher *NQO2* levels than its median expression in healthy individuals, which is statistically significant in the analyzed population, characterized by nonnormal distribution. Considering a high degree of heterogeneity among PD patients^4^ as well as the presence of polymorphisms leading to high or low *NQO2* expression, such a non-Gaussian distribution is plausible for both healthy and PD populations. We do not know if the levels of blood-born transcripts predict the brain expression of a given gene, but higher NQO2 expression in the blood may indicate a more active promoter. According to our analysis, elevated *NQO2* expression is almost 3 times more frequent among PD patients than in the control group, which is in line with the observations of the study by Harada et al.^35^.

In conclusion, our observations suggest that drugs targeting NQO2 may stimulate autophagy as well as protect against dopamine quinone and hydroxyquinone toxicity. Thus, S29434 and other NQO2 inhibitors by acting as protective agents in astrocytes, might delay neuronal damage in PD and other pathological conditions involving oxidative neurodegeneration. The vast literature suggesting a protective effect of several natural polyphenols in neurodegeneration models strongly supports this idea, since NQO2 is a well-documented target of these compounds^40,67^. Further studies in PD animal models and brain organoids will be necessary to validate this interesting hypothesis.

## Methods

### Reagents and antibodies

Unless otherwise specified, all reagents were purchased from Sigma-Aldrich/MERCK (Darmstadt, Germany): 6OHDA (H4381, 100 mM stock in bi-distilled H2O (H2Ob)), paraquat (M2254; 100 mM stock in H2O), and chloroquine (ClQ, C6628; 25 mM stock in H2Ob). Except for 6OHDA, which was freshly prepared for each experiment and stored at − 80 °C for another use within one week, other reagents were kept in aliquots at − 80 °C for longer times and thawed shortly before each treatment. S29434, previously well characterized^39^ and used under the name of NMDPEF^31,32^ was stored as a 10 mM stock in DMSO (D4540) at − 80 °C. The antibodies (Abs) used for Western blotting (WB) were: polyclonal rabbit anti-LC3A/B (Abcam, ab1280025 used 1:2000), anti-p62 (MBL, PM045 used 1:1000), anti-GAPDH, clone FL335 (Santa Cruz Biotech., sc-25778 used 1:200), anti-NQO2 (ProteinTech, 15767-1-AP used 1:1000) and monoclonal mouse anti-α-Tubulin (Sigma-Aldrich, T6074 used 1:500).

### Cell culture

Astrocytoma U373 cells were a kind gift from M. Pollicita and S. Aquaro, Tor Vergata University, Rome. After thawing, cells were expanded in high glucose (4.5% glucose) Dulbecco’s modified Eagle’s medium (DMEM) (Carlo Erba S.r.l., Milan, Italy, FA30WL0101500) and then maintained in these conditions for routine culture or gradually switched to low-glucose (1% glucose) DMEM (FA30WL0060500) when indicated. Each type of DMEM was supplemented with 10% fetal bovine serum (FBS) (Life Technologies, Monza MB, Italy, 10500-064), 1:100 penicillin–streptomycin (Pen Strep, Life Technologies, 15070-063), and 1:100 l-glutamine (PAA Laboratories GmbH, Austria, M00409-2722) to constitute a standard medium, and cells were cultured under standard 5% CO2 conditions. If not otherwise specified for most experiments, U373 cells were seeded 2 days before or 3 days before the treatments at a density of 32 × 10^3^ or 16 × 10^3^ × cm^−2^ (300 or 150 × 10^3^ per 3.5 cm plate), respectively, and the last medium change was performed 24 h before the treatment with a desired compound. For longer treatments (48 h and 72 h), 6OHDA was re-added every 24 h, and the medium change was performed every 48 h. ClQ was added 2 or 3 h before cell lysis as indicated. The stressful culture conditions were generated by switching the cells cultured for one week in high glucose to low glucose or by seeding them at low density (6.4 × 10^3^ × cm^−2^) for 2 days before treatments. Neuroblastoma SH-SY5Y cells were cultured in F-12 K medium plus high glucose DMEM supplemented with 10% FBS, Pen Strep and l-glutamine as standard medium.

### ROS detection in live cells

ROS were detected by two alternative ROS fluorescent indicators: a dichlorofluorescein (DCF) derivative, namely, 5(6)-carboxy-2′,7′ dichlorofluorescein diacetate (CA-DCF-DA; Sigma-Aldrich, 21884) or MitoSOX (3,8-phenanthridinediamine, 5-(6′-triphenylphosphoniumhexyl)-5,6 dihydro-6-phenyl). The first method detected cytoplasmic ROS in live cell monolayers by fluorimeter, while the second method detected ROS in trypsin-detached cells by FACS analysis. The DCF-based method was performed as previously described^31^, with minor modifications. U373 cells were plated at 2 × 10^4^/cm^2^ in 96-well plates 2 days before treatments, but phenol-free DMEM (Gibco, Life Tech. 11054020) was used as the basal medium. 6OHDA was added 6 h before analysis in triplicate for each concentration. Cell staining with CA-DCF-DA was performed 2 h before analysis. For this purpose, the conditioned medium +/− 6OHDA was temporarily removed, and the cells were incubated with prewarmed basal phenol-free DMEM (Gibco, Life Tech. 11054020) containing 20 mM CA-DCF-DA or vehicle (DMSO) for 30 min. Stained cells were rinsed in phenol-free DMEM and incubated again for 1.5 h with the conditioned medium. All medium changes and washes were performed gently to avoid any shear, osmotic or other types of stress, drop by drop placed on the plate wall with the warm medium. Fluorescein signal detection of cell monolayers was performed on a Victor 2 multilabel fluorimeter (Perkin-Elmer, Singapore). Background readings (cells stained with DMSO) were subtracted for the analysis of fluorescence of DCF-DA-stained cells.

For detection of mitochondrial ROS, U373 cells were plated at 1.2 × 10^4^ cells/per well on 24 well plates in low-glucose DMEM standard medium, 2 days before treatment. MitoSox was used at a concentration of 5 µM in phenol-free DMEM.

### Western blotting (WB)

See Supplementary Methods.

### NQO2 activity measurements

NQO2 enzymatic activity assays were performed as described previously^40^ with the following modifications. Cellular NQO2 activity was measured in U373 cell lysates. For this purpose, the cells were cultured for two days to reach a 70–90% density and then treated with 6OHDA for 24 h, scraped and homogenized in ice-cold GPS buffer (50 mM Tris–HCl, 3 mM n-octyl-d-glucopyranoside pH 7.5) with 10 strokes by a tight glass douncer on ice. After clearing by centrifugation, the lysates were stored at − 80 °C. After thawing, lysates (5 mL containing 8 to 15 mg protein) were aliquoted on 96-well plates placed on ice and then equilibrated for 10 min at RT. The reaction was initiated by the addition of GPS buffer supplemented with 50 mM of the cosubstrate 1-benzyl-1,4-dihydro-nicotinamide (BNAH; Santa Cruz Biotech., 208609) with or without 50 mM menadione (K3) (Supelco, Merk Life Science S.r.l., Milan, Italy) as substrate in a total volume of 200 mL. Testing of 6OHDA as an NQO2 substrate was performed with 50 or 100 ng/mL human recombinant NQO2 (Sigma-Aldrich, Q0380) in GPS buffer plus 100 mM BNAH and different concentrations of 6OHDA or 100 mM K3 as a positive control in a total volume of 200 mL. BNAH supplemented with an appropriate amount of NQO2 and vehicle instead of 6OHDA was used as a negative control. The reaction was initiated by the addition of the recombinant enzyme to each column of 96-well plates. The exact time of pipetting the enzyme (time 0) was taken to calculate the delay between the fluorescence reading and the reaction start. BNAH fluorescence was monitored by a 1420 Multilabel reader Victor 2 fluorimeter (Perkin Elmer) for 15 to 20 min. Data were analyzed by Microsoft Excel and GraphPad Prism 9.0 software (GraphPad, San Diego, CA, USA) to fit the decay curves and find unknown values by curve interpolation. The velocity (V) of the reaction was determined from the fluorescence change as the mean reduction of mmol of BNAH (in 200 mL) per minute for the first 5 min after the addition of the enzyme. The activity was calculated as V divided by the enzyme/protein amount added to the reaction.

### Preparation of NQO2-overexpressing (N-over) U373 cells

See Supplementary Methods.

### Flow cytometry (FACS) analysis of cell viability

See Supplementary Methods.

### Coculture of astroglial and dopaminergic cells

U373 cells and U373 N-over cells were seeded and cultured on 24-well plates in high-glucose DMEM. Briefly, U373 cells were plated at a density of 40 × 10^3^ cm^−2^, while U373 N-over cells were plated at a density between 30 × 10^3^ cm^−2^ under standard conditions to achieve a 90% confluent monolayer after 2 days. Before plating, SH-SY5Y cells were labeled with CellTracker Green CMFDA (chloromethyl fluorescein diacetate, Molecular Probes, Life Tech., USA) dye. Briefly, SHSY5Y cells, grown to 80–90% confluency, were labeled with 5 µM CMFDA in PBS for 15 min. Cells were washed once in PBS, and fresh regular medium was added for 30 min. Finally, the cells were trypsinized (Trypsin–EDTA (0.05%), Gibco, Life Tech., USA), and resuspended in fresh culture medium to a desired cell concentration. Next, 5 × 10^4^ cm^−2^ SH-SY5Y cells were seeded over U373 monolayers. After 24 h, the cocultures were treated for 48 h with 6OHDA and/or S29434. At the end of the treatment, the cells were washed once in PBS (Carlo Erba S.r.l., FA30WL0615500), trypsinized for 3 min and collected in 0.5 ml of F12-K medium plus high glucose supplemented with 10% FBS in flow cytometry round tubes. The cells were then pelleted by centrifugation for 5 min at 1000 × *g* and resuspended in 400 µL of PBS containing 1% FBS and 0.5 mM EDTA. Cell viability and mortality were determined by 7-AAD dye (BD Biosciences, Erenbodgem, Belgium, cat. nr 344563; used 1:10) added 2–3 min before FACS analysis on a FACSCanto II flow cytometer (BD Biosciences, Erenbodgem, Belgium). The data were acquired in the FITC channel (for CMFDA dye) and PerCP-Cy5-5 channel (for 7-AAD dye) according to the parameters and gating criteria detailed in the Supplementary Methods and Fig. S4 (Supplementary information link).

### U373 cell transfection and silencing of NQO2

U373 cells cultured in high glucose standard medium were seeded on 6-well plates one day before transfection at a density of 4 × 10^4^ cells cm^−2^. Nonsilencing control siRNA [*siEGFP*, (EHUEGFP)] and a set of human *NQO2*-targeting *esiRNAs* (EMU042521) were purchased from Merck, Sigma-Aldrich. Transfection of U373 cells was performed using Transfection Reagent (Merck, S1452) and Opti-MEM (Life Technologies, 51985026) according to the manufacturer's instructions. Briefly, siRNA (150 nM) and transfection reagent (1:1 ml/pMol siRNA) were diluted in Opti-MEM and incubated for 15–20 min at RT. Cells were incubated in Opti-MEM containing siRNA-transfection reagent complexes for 4 h and then incubated in high-glucose culture medium containing w/o PenStrep. After 20 h, the medium was exchanged for regular high-glucose medium. The treatments were initiated 48 h after transfection and carried for the next 24 h. ClQ (25 µM) was added 3 h before the end of the experiment. After the treatments, the cells were lysed and assayed by WB.

### Animal procedures

*NQO2* knock-out mice generated in the C3H/HeOuJ background were previously characterized^38,68^. Since the first description in 2004, the original knockout mice were backcrossed with *WT* C3H/HeOuJ mice obtained from Charles River at least 20 times. Mice used for astrocyte isolation were WT and *NQO2* − / − pups of the C3H/HeOuJ strain born on the same day from the same litter *WT* and *NQO2* − / − females crossed with the same litter males. One- to two-day-old (P1/P2) pups were euthanized by head decapitation. The heads were placed into sterile PBS at RT, and the meninges were carefully removed. The animal procedures were carried out according to EU Directive 2010/63/EU and approved by the Italian Ministry of Health (authorization Nr. 613-2017-PR). This study complied with ARRIVE guidelines (https://arriveguidelines.org/).

### Isolation and culture of mouse cortical astrocytes

Primary astrocytes were isolated from cerebral cortices prepared as described above and according to a previously published protocol^31^. Briefly, the cortices were transferred to new dishes with sterile PBS and then dissected, minced, and mechanically dissociated. Minced cortices were centrifuged (800 g, 8 min, 4 °C), and the pellet was digested in DMEM containing 10 mg/mL type I DNase for 15 min on a benchtop orbital shaker (100 RPM) at RT. The reaction was stopped by adding ice-cold DMEM, and the homogenate was pelleted by centrifugation (800 g, 8 min, 4 °C). The cells were plated on 10 cm dishes (1 brain/dish) in DMEM supplemented with 10% FBS and 1% P/S (Gibco, Life Tech.). The next day, the medium was changed to remove non-adherent cells. Thereafter, the medium was changed every 2 days. The cultures were maintained for approximately 1 week or until 90% confluent at 37 °C and 5% CO_2_. Cells were then removed from the dish with the addition of 0.05% trypsin–EDTA (Invitrogen) and replated on 6-well plates for experiments at a density of 500,000 cells per well. The treatments were performed one day after seeding.

### Phase-contrast and fluorescence microscopy

Phase-contrast and fluorescence images were acquired with LEICA DMI 4000 B fluorescent microscope, equipped with LEICA DFC 350 FX camera.

### Microarray data information and Gene Expression Omnibus (GEO) dataset analysis

See Supplementary Methods (Supplementary information link).

### Data analysis and statistical procedures

Numerical data from all multigroup experiments were evaluated by analysis of variance (ANOVA). If Brown-Forsythe’s test for equality of variances was positive, Welch’s ANOVA test was performed, otherwise ordinary one-way ANOVA or occasionally three-way ANOVA was performed. Multiple comparisons between groups were performed by one of the suggested posttests according to GraphPad PRISM 9.0 software guidelines http://www.graphpad.com/ or occasionally by unpaired T test as indicated in figure legends. Data are expressed as the means ± SEM for the number n of independent experiments or as the means ± SD for the number of n independent samples from a representative experiment, as indicated for each figure. For patient and healthy subject expression data, D'Agostino-Pearson and Shapiro–Wilk normality tests were performed. If the data did not pass the normality tests or groups were lower than 10, Mann–Whitney test was applied and the data were presented as medians +/− range; otherwise unpaired T test was used.

### Ethics declaration

The authors declare that the current study complied with ARRIVE guidelines (https://arriveguidelines.org/).

### Supplementary Information

Supplementary Information.

## Data Availability

The original images of blots presented in this work are displayed in the *Supplementary Information*. All the other datasets generated and analyzed during the current study are available from the corresponding author upon request.
