# Supplementary material for: Autophagy and neuroprotection in astrocytes exposed to 6-hydroxydopamine is negatively regulated by NQO2: relevance to Parkinson’s disease
Source: Sci Rep. 2023 Dec 7;13:21624. doi: 10.1038/s41598-023-44666-7 (PMC10703796; doi:10.1038/s41598-023-44666-7)
Supplement: Supplementary file 1 — Supplementary Information. [file 41598_2023_44666_MOESM1_ESM.pdf]

Supplementary Information

Autophagy and neuroprotection in astrocytes exposed to 6-hydroxydopamine is negatively regulated by NQO2: a potential relevance to Parkinson's disease.

Elzbieta Janda[1*], Maddalena Parafati[1,2§], Concetta Martino[1§], Francesco Crupi[1], Jonahunnatha Nesson George William[3], Karine Reybier[4], Mariamena Arbitrio[5]*, Vincenzo Mollace[1] and Jean A. Boutin[4].

[1] Department of Health Science, Laboratory of Cellular and Molecular Toxicology, University "Magna Græcia" of Catanzaro, 88100 Catanzaro, Italy

[2] Current address: Department of Pharmacodynamics, University of Florida, USA

[3] Center for Advanced Studies and Technology (CAST), "G. d'Annunzio" University of Chieti-Pescara, Chieti, Italy.

[4] UMR 152 Pharma-Dev, Université de Toulouse III, IRD, UPS, 31400 Toulouse, France

[5] Institute for Biomedical Research and Innovation (IRIB), National Research Council of Italy (CNR), 88100 Catanzaro, Italy

[§] Both authors contributed equally to this work.

[*]Correspondence : janda@unicz.it, mariamena.arbitrio@cnr.it

Supplementary Figures

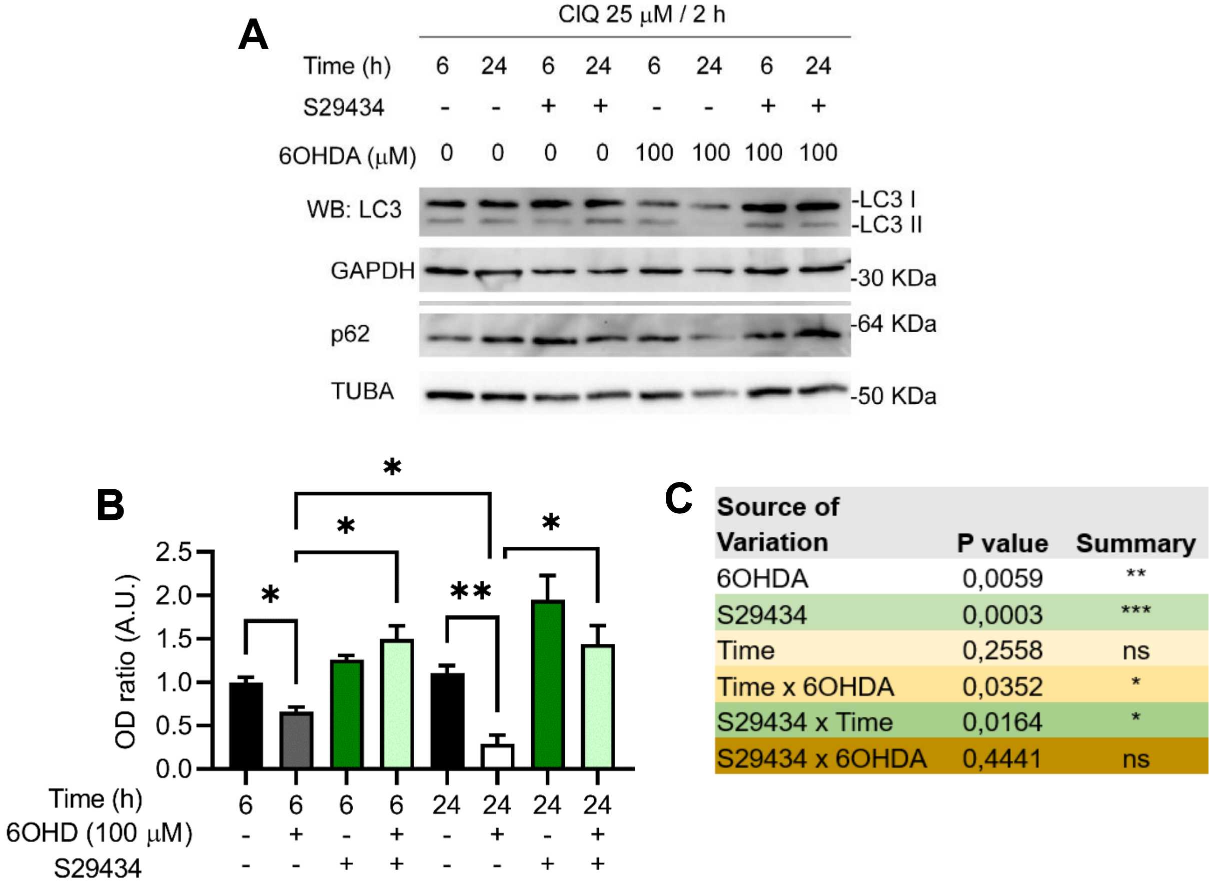

Source of Variation	P value	Summary
6OHDA	0,0059	**
S29434	0,0003	***
Time	0,2558	ns
Time x 6OHDA	0,0352	*
S29434 x Time	0,0164	*
S29434 x 6OHDA	0,4441	ns

Fig. S1. Time-dependent downregulation of autophagic flux in response to 6OHDA in U373 cells. A) LC3II levels were analyzed in U373 astrocytes, cultured in optimal conditions and exposed to 100 µM 6OHDA +/- S29434 (10 µM) for 6 or 24h. ClQ (25 µM) was added to all samples for 2 h before lysis and protein lysates were analyzed for LC3, NQO2 and p62 levels by 12% and 8% SDS-PAGE respectively. GAPDH and α- Tubulin were used as loading controls for LC3 and p62, respectively. **B**) Densitometric analysis of 3 independent experiments performed as described in A and B. Bar +/- SEM. Statistical analysis: One-way ANOVA followed by unpaired t with Welch's correction test; *P < 0.05, **P < 0.01 or by three-way ANOVA as shown in **C).**

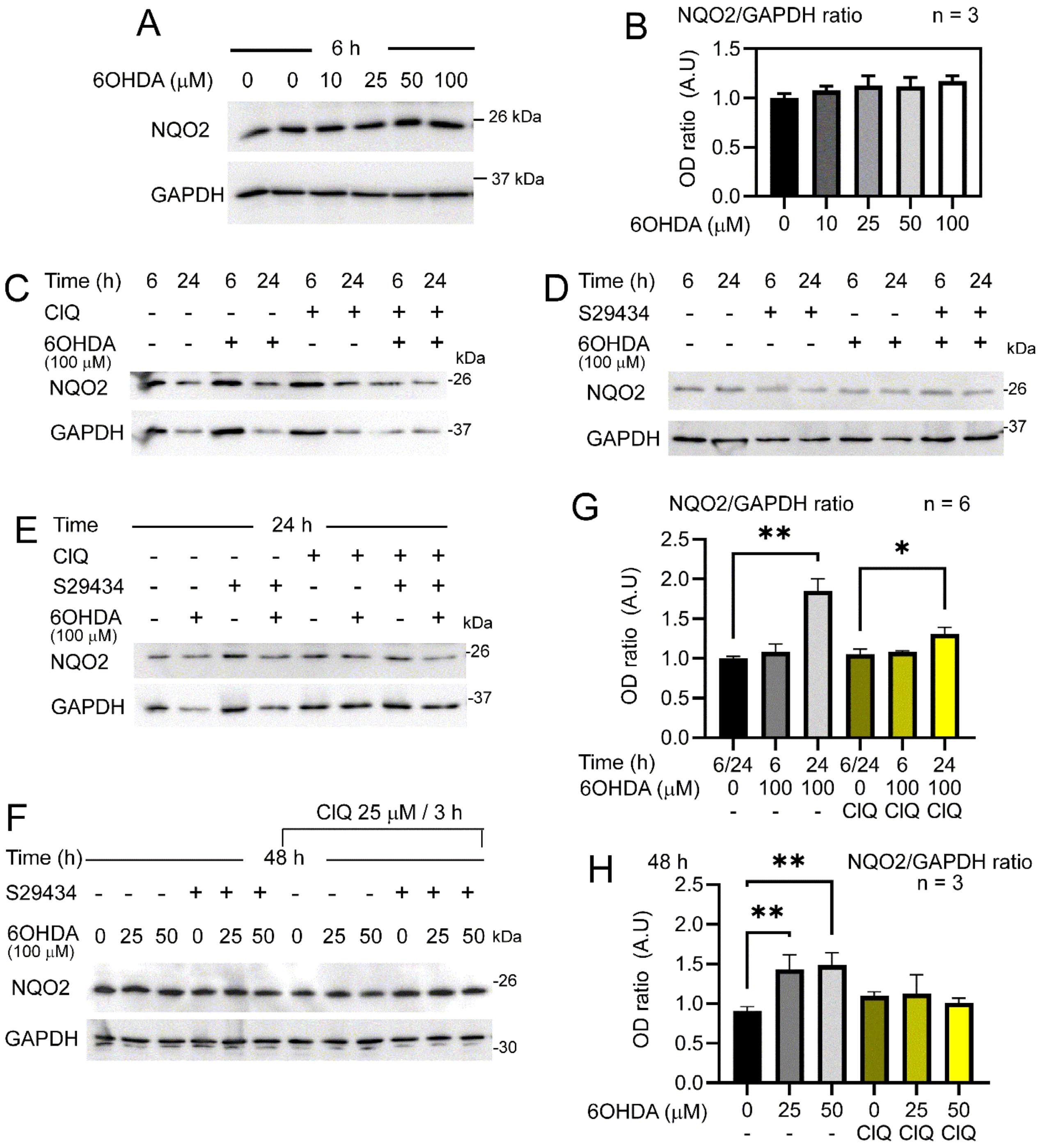

Fig. S2 Time-dependent upregulation of NQO2 expression levels by 6OHDA. A, **C-E**) The effect of 6OHDA on NQO2 levels was analyzed by 15% or 12% SDS-PAGE and WB in different experiments comparing the time of exposure and the impact of the co-treatment with ClQ, including those described in Fig. 1 and S1, after blotting with anti-LC3 antibody. U373 astrocytes were plated under standard conditions and GAPDH was used as a loading control in all experiments described below. A) Cells were exposed to different concentrations of 6OHDA for 6h before cell lysis. **B)** Densitometric (OD) analysis of n = 3 independent experiments described in A. One-way ANOVA followed by Tukey post-test: not significant (ns) for all comparisons. **C)** Cells were exposed to 100 μM 6OHDA for 6 or 24 h +/- ClQ (25 μM). ClQ was added for 2 h before cell lysis and protein lysates were analyzed for NQO2. **D)** Cells were treated with 100 μM 6OHDA +/- S29434 (10 μM) for 6 and 24 h as in the experiment described in Fig. S1. **E)** Cells were exposed to 100 μM 6OHDA +/- S29434 (10 μM) and +/- ClQ (25 μM) added as in the experiment described in Fig. 1C. **F)** NQO2

levels were analyzed in cells treated with 25 or 50 µM 6OHDA +/- S29434 (10 µM) for 48 h, +/- ClQ (25 µM) added for 3 h before cell lysis. **G)** OD analysis of NQO2 to GAPDH signals ratio from 5 independent experiments described in C to E. Statistical analysis: Welch ANOVA followed by Unpaired t with Welch's correction post-test; *P < 0.05, **P < 0.01. **H)** OD analysis of NQO2 to GAPDH signals ratio from 3 independent experiments described in F. Statistical analysis: One-way ANOVA followed by Sidak's post-test. Bars in B, G and H show the mean +/- SEM normalized to the mean OD ratio of 2 different reference bands on the same blot (f.e. controls 6 and 24 h).

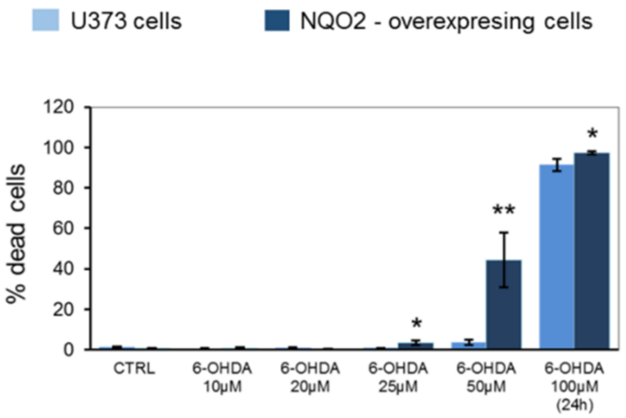

Fig. S3. NQO2 overexpression sensitizes U373 cells to 6OHDA-induced toxicity. Empty vector U373 (Control) cells and NQO2 overexpressing (N-over) U373 cells were plated in DMEM-high glucose and exposed to different concentrations of 6OHDA for 48h. Cell mortality was analyzed by trypan blue exclusion assay and flow cytometry. The graph shows the mean +/- S.D. of a representative experiment out of 3 independent experiments, performed in triplicate. Statistical analysis: unpaired, 2-tailed t test (t-test); *P < 0.05, **P < 0.01.

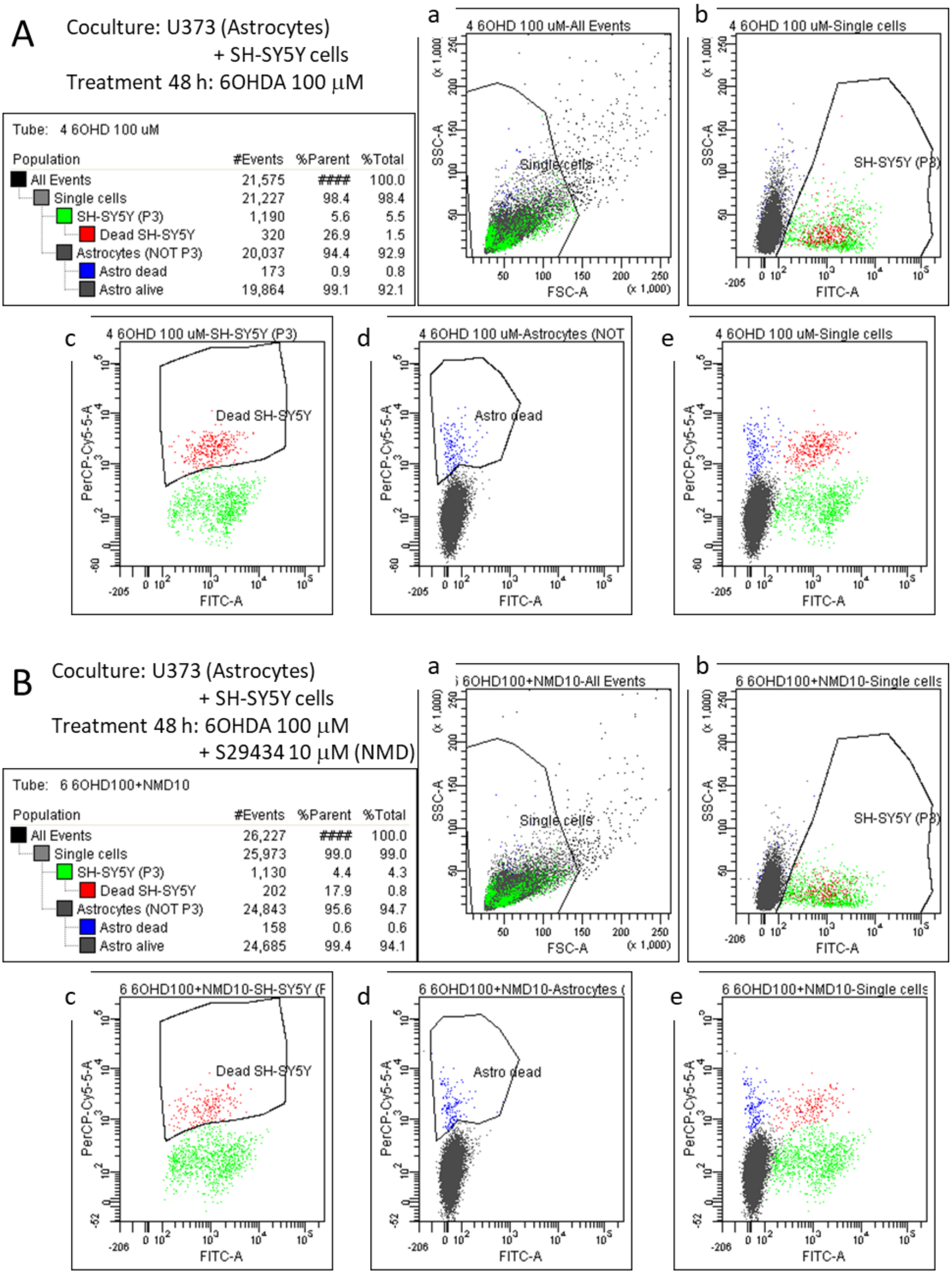

Fig. S4 Gate selection for flow cytometry in the coculture experiments. The gating criteria applied for the experiments shown in the main Figure 6 are presented on two examples of samples with the coculture of U373 (Astrocytes) and SH-SY5Y cells: A) treated 48 h with 6OHDA (100 μM); B) treated 48 h with 6OHDA(100 μM) and S29434 (10 μM). Dot-plots (a – e) are described in Supplementary Methods below.

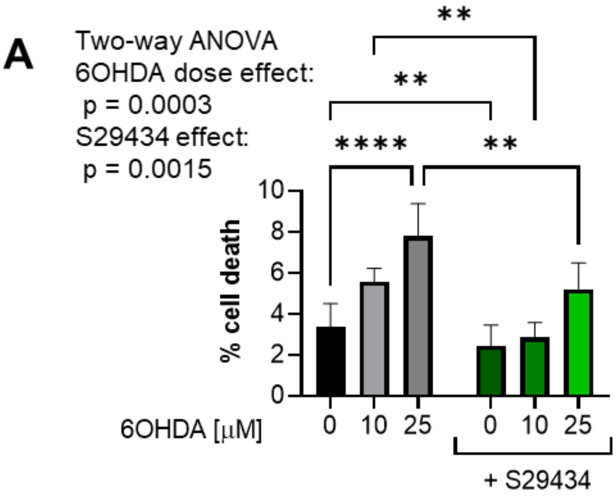

B

6OHDA conc.	ΔA % cell death DMSO	ΔB % cell death S29434	% Protect. S29434	SD
0	0	-0.9	128	43.1
10	2.2	-0.5	109	29.6
25	4.4	1.8	58.6	20.8
50	15.6	8.1	48.1	22.0
100	30	16.6	44.4	13.5

Figure S5. **The relative protection from 6OHDA-induced toxicity exerted by S29434 in coculture with U373 cells is dependent on the concentration of 6OHDA.** SH-SY5Y cells labeled with CMFDA (green) were added to U373 cell monolayers. After 24 h of coculture, cells were exposed to 6OHDA +/- 10 µM S29434 for the next 48 h. Cell mortality was analyzed by flow cytometry after 7-AAD staining in green cell population (SH-SY5Y cells). A) The analysis of cell death in SH-SY5Y cells from a representative experiment performed in triplicated. Bars show mean % +/- SD. Statistical analysis: Two-way ANOVA followed by uncorrected Fisher's LSD test; **p < 0.01, ***p < 0.001, ****p < 0.0001. B. The relative % cell death (Δ) was calculated as the difference in % cell death between cells exposed to 6OHDA and the background mortality of SH-SY5Y cells in coculture (% cell death in cells not exposed to 6OHDA), which is around 3,4%. ΔA and ΔB are % cell death in cells treated with 6OHDA minus 3.4% and in cells treated with 6OHDA and S29434 minus 3.4%, respectively. The protection exerted by S29434 on SH-SY434 cells was calculated from the formula: 100-(ΔB/ΔAx100), thus it represents % reduction in relative cell death (after subtracting 3.4%) in response to S29434.

Supplementary Methods

Flow cytometry (FACS) analysis of cell viability

U373 cells were plated at standard density ($3{,}2 \times 10^4$ cells cm^{-2}) on 24 well plates in high- or low-glucose medium or at 5-times lower density ($0{,}64 \times 10^4$ cells cm^{-2}) two days before the first treatment. After 72 h, growth medium was removed the cells were washed with PBS (pH 7.4) and trypsinized for 3–4 min, (trypsin 0.05%), neutralized in pre-warmed regular medium and collected in FACS tubes. The cells were then pelleted by centrifugation for 5 min at 1000 x g and resuspended in 450 µL PBS containing 1% FBS and EDTA 0.5 mM. Trypan blue solution in PBS (0.016%; 100 µL) was added 1– 2 min before FACS analysis (FACSCanto II, BD Biosciences, Erenbodgem, Belgium). The data were collected in 630 nm (PerCP-Cy5-5) channel.

Flow cytometry (FACS) analysis of cell viability in coculture experiments

For the coculture experiments SH-SY5Y cells were stained with CMFDA dye, seeded on monolayers of control U373 or N-over U373 (overexpressing NQO2) cells, treated with 6OHDA +/- S29434 for 48 h and collected as described in the main manuscript. The populations were selected by using the following acquisition parameters on FACSCanto II (BD Biosciences, Erenbodgem, Belgium) flow cytometer: Main channels and voltages: FSC channel: 285, SSC channel: 400, FITC channel: 350, PerCp-Cy5-5: 370. Voltages were adjusted for FSC and SSC, FITC and PerCp-Cy5 channels, but other channels were also active at low voltage. Cut-off threshold was 20,000. The compensation of channels was not necessary. The population gates were selected in 5 steps. Step 1: Selection of the "single cells" gate as shown in FSC/SSC scatter plots (a) in Figure SA,B) and confirmed in FSC-A vs FSC-H and FSC-A vs FSC-W. Step 2: Selection of green P3 (FITC-positive) gate within "single cells" gate corresponding to cells labelled with CMFDA (chloromethyl-fluorescein diacetate, Molecular Probes, Life Technologies, U.S.A.) dye and denominated as SH-SY5Y cells (C-Fb) and the definition of the opposite gate (not P3) as "Astrocytes". Step 4: Selection of 7-AAD positive within SH-SY5Y cells denominated as Dead SH-SY5Y cells. Step 5: Selection of 7-AAD positive cells within "Astrocytes" gate denominated as Dead Astrocytes.

Western Blotting (WB)

At indicated times after the treatments, cell monolayers were washed once with pre-warmed phosphate-buffered saline (PBS) (Carlo Erba srl, Milan, Italy, FA30WL0615500) and the proteins were extracted with ice-cold lysis buffer containing 50 mM Tris-HCl, pH 7.3, 150 mM NaCl and the following reagents from Sigma-Aldrich (Merk, Sigma-Aldrich, Darmstadt, Germany): 1% Igepal (I7771), 1 mM EDTA (E5134), phosphatase inhibitors (1mM NaF [S1504], 0.01 mM sodium orthovanadate [S6508], 10 mM β-glycerophosphate [G9422] and protease inhibitor cocktail (Mini tablets, Thermo Fisher Scientific, Waltham, MA, USA, 88665) and subsequently processed as previously described [30]. Samples containing similar amounts of protein (10–25 µg) were boiled for 3 min at 90°C before loading onto 15% or 8% polyacrylamide SDS (SDS-PAGE) gels and transferred to polyvinylidene difluoride (PVDF) membranes (Bio-Rad Laboratories S.r.l, Milan, Italy 162–0177) and WB was performed, developed and digitally acquired with Molecular Imager ChemiDoc XRS (Bio-Rad), as previously described [30, 39]. The molecular weight of examined proteins were estimated by electrophoresis of *All Blue*™ or *Kaleidoscope* Precision™ Plus Protein Standards, (Bio-Rad, 161-0373 or 161-0375) or *PINK* Pre-stained™ Protein Markers (Nippon Genetics Europe, Duren, Germany, MWP02) or Fisher Bioreagents™ *EZ-Run*™ Prestained *Rec* Protein Marker (Thermo Fisher Scientific, BP3603-1).

To evaluate the results of WB, relevant bands were quantified by Quantity One® 1-D image analysis software (Bio-Rad) and the volume optical density (OD) data were first divided by OD of corresponding loading controls and then normalized to the means of two control bands. These means were assigned an arbitrary value of "1" for comparison with other independent blots. All WB experiments were replicated at least 3 times.

Preparation of NQO2-overexpresing cells

NQO2-overexpressing U373 cells (N-over) were generated by infection with a lentivirus coding for human NQO2pLenti6.2# × 2044; V5-DEST™ Gateway® as previously described [30]. The aliquots of N-over cells were kept frozen and used for experiments after thawing, followed by 4 days reselection in blasticidin (Invitrogen, Life Technologies (Tech.), Road Gran Island, NY, USA) and 4 d recovery in the absence of the drug. Due to the faster growth rate of N-over cells with respect to control vector U373 cells, N-over cells were plated at 30 - 40 % lower density (20 x 10^3 cm^{-2}) 2 days before treatments than the later cells under standard conditions to achieve the same density (70 – 80 % confluent monolayer) after 2 days.

Microarray data information and Gene Expression Omnibus (GEO) datasets analysis

The gene expression profile GSE6613, GSE8397 and GSE100054 were obtained from NCBI-GEO Datasets. The microarray data of GSE6613 are based on GPL96 platform, (HG-U133A Affymetrix Human Genome U133A Array) and include purified whole blood samples from 50 patients with Parkinson's disease, 33 with neurodegenerative diseases other than PD, and 23 healthy controls. GSE8397 is based on GPL96 and GPL97 platforms (HG-U133A/B Affymetrix Human Genome U133A Array) and include a total of 47 individual purified tissues samples for each gene chip per sample comprising 15 medial parkinsonian *substantia nigra* (SN), 9 lateral parkinsonian SN, 8 medial SN control and 7 lateral SN control samples. Lateral and medial SN samples were from the same cases. In addition, frontal cerebral cortexes in 5 of the PD cases and in 3 of the controls. The GSE100054 is based on GPL23126 Affymetrix Human Clariom D Assay and include purified PBMC samples from 9 normal controls and 10 patients with PD. In GSE6613, GSE8397, the *NQO2* (Gene ID: 4835) gene expression was analyzed by its probe 203814_s_at in GPL96 platform, long non -coding (ln) RNA [NQO2] was analyzed by its probe 237870_at in GPL97 platform and in GSE1000054, NQO2 is analyzed by its probe TC0600014055.hg.1 in in GPL23126 platform. The obtained datasets from GEO repository were not subjected to any further normalization since they had been processed and normalized to become cross-comparable.

Supplementary Images

Raw Western blot images

In the section below the raw Western blot images are presented, which were used for the preparation of all main (Fig, 1A, C, E; Fig. 5A, C, E) and supplementary figures (S1A, S2A, S2C-F). In some cases, additional whole protein staining (Amid black) blots were presented. All images are described with figure references, antibody or staining, code of the gel and % acrylamide (in green) and molecular weight ladders (in kDa), according to used commercial protein markers. Red arrows indicate specific bands.

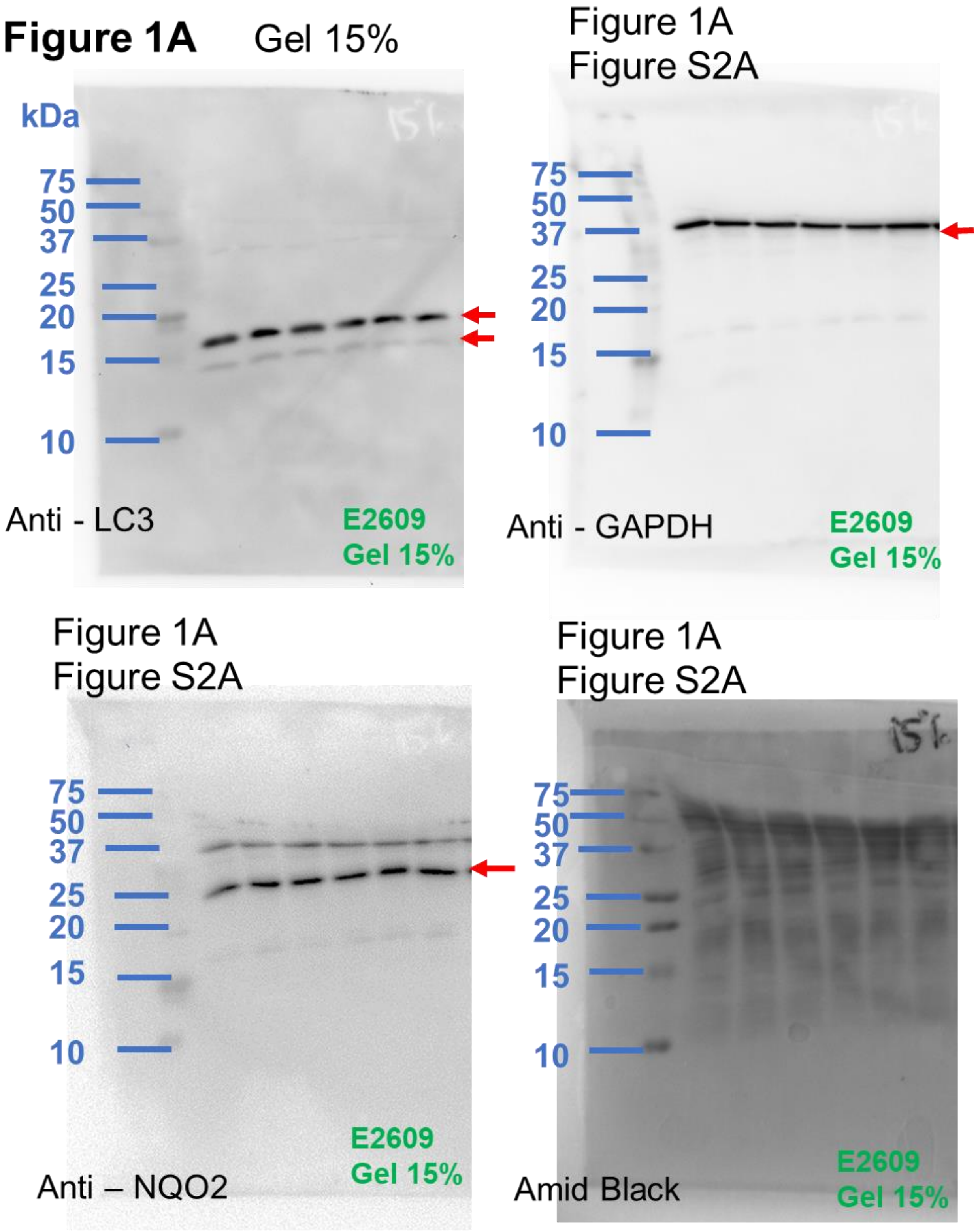

Precision Plus Protein
Kaleidoscope Marker,
Biorad
Cat. No. 1610375

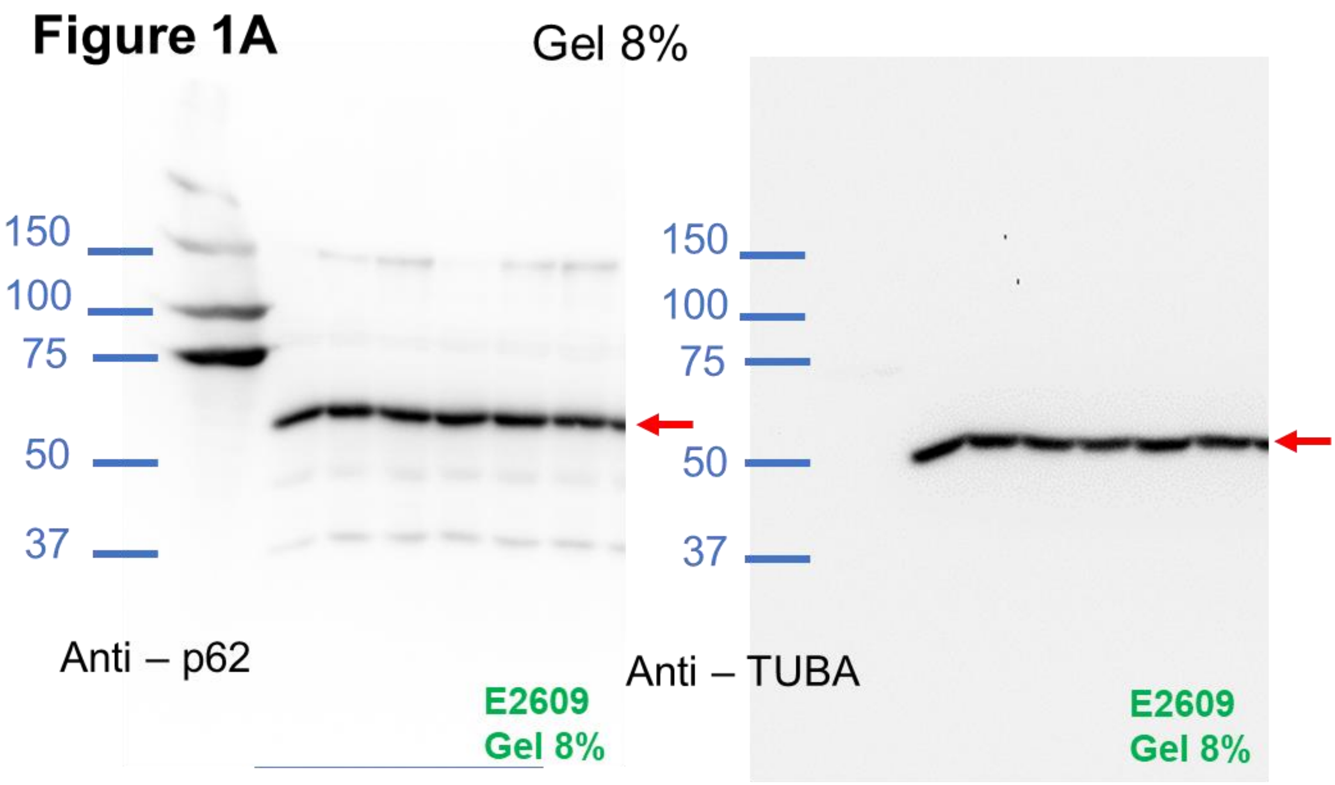
Figure 1A
Gel 8%
150
100
75
50
37
Anti – p62
E2609
Gel 8%
150
100
75
50
37
Anti – TUBA
E2609
Gel 8%

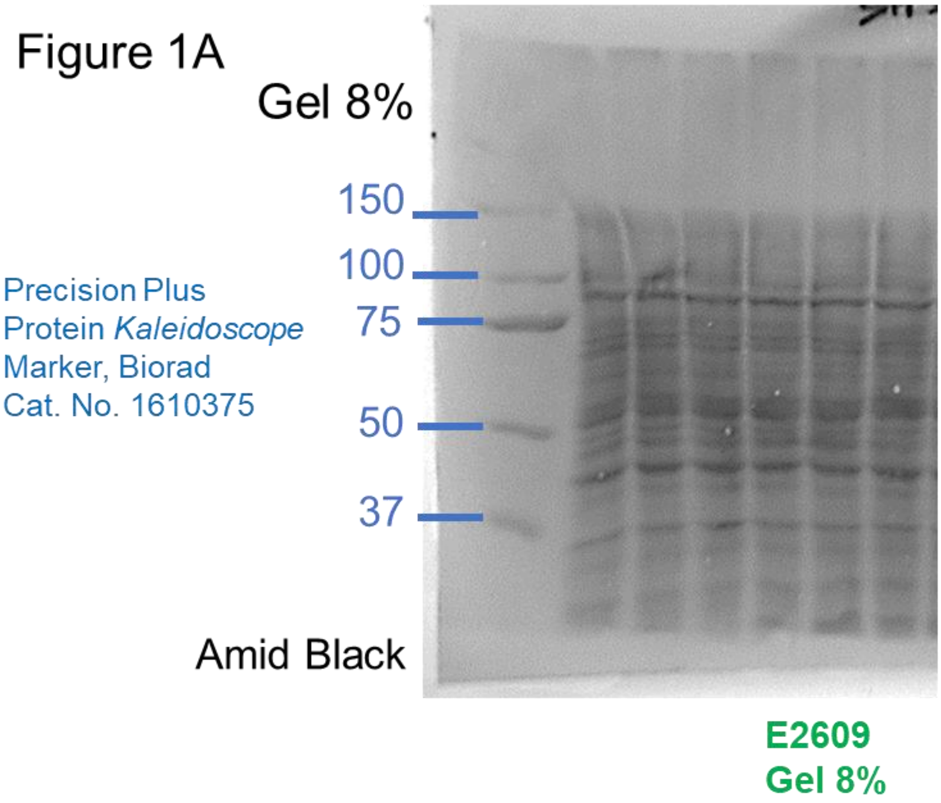
Figure 1A
Gel 8%
150
100
75
50
37
Precision Plus
Protein *Kaleidoscope*
Marker, Biorad
Cat. No. 1610375
Amid Black
E2609
Gel 8%

Figure 1A

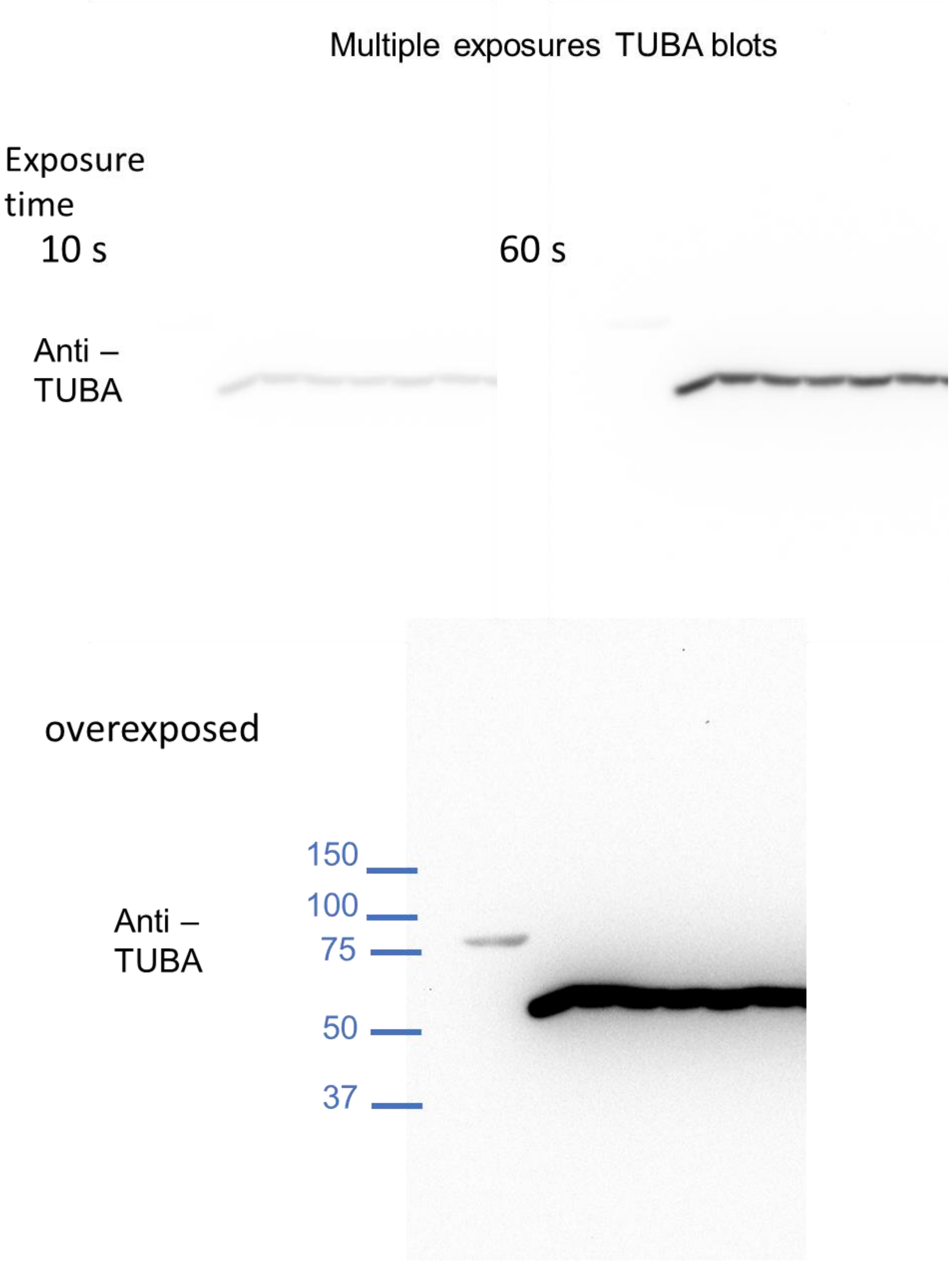
Multiple exposures TUBA blots
Exposure time
10 s
60 s
Anti – TUBA
overexposed
150
100
75
50
37
Anti – TUBA

Figure 1C

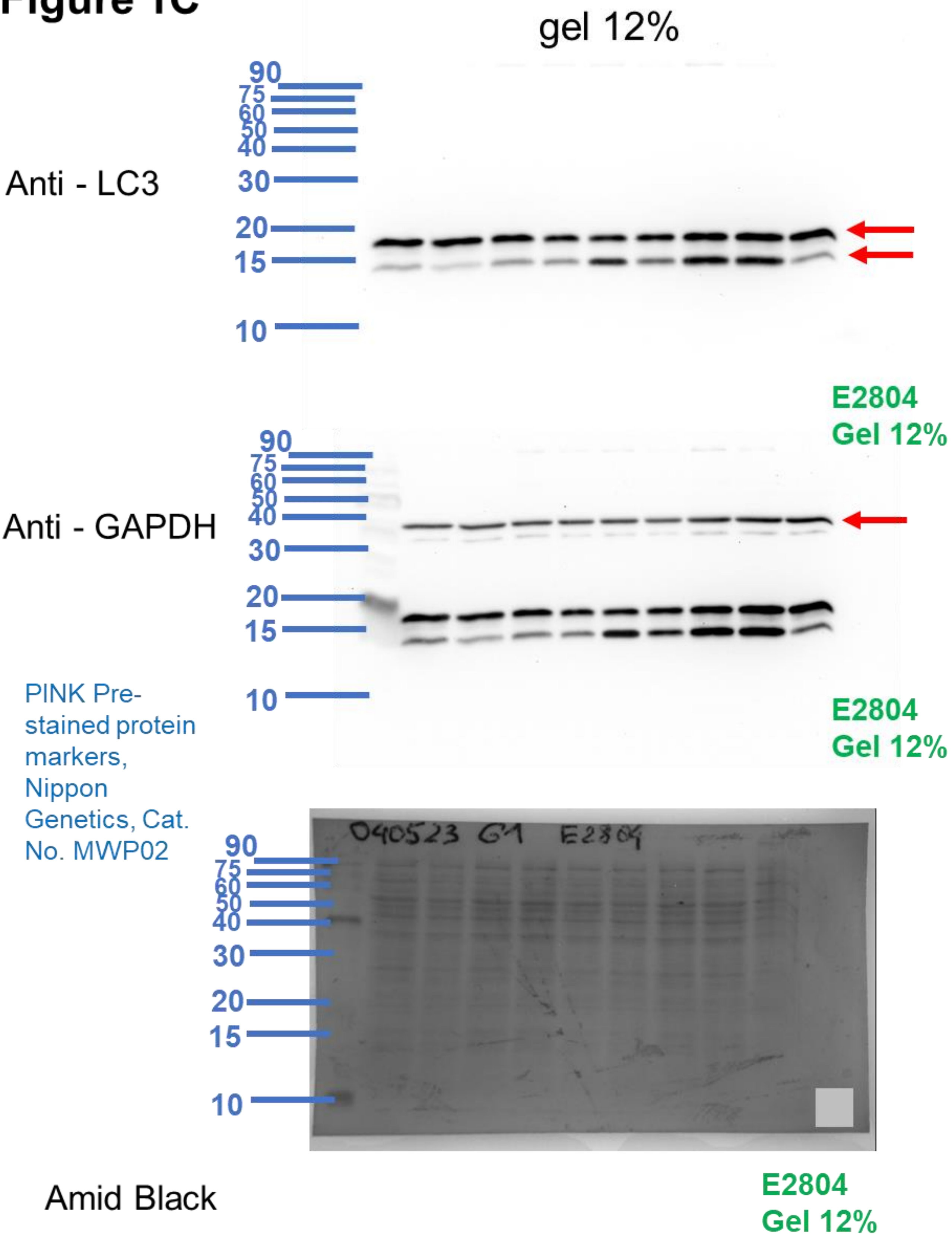
gel 12%
Anti - LC3
90
75
60
50
40
30
20
15
10
E2804
Gel 12%
Anti - GAPDH
90
75
60
50
40
30
20
15
10
E2804
Gel 12%
PINK Pre-stained protein markers, Nippon Genetics, Cat. No. MWP02
90
75
60
50
40
30
20
15
10
Amid Black
E2804
Gel 12%

Figure 1C

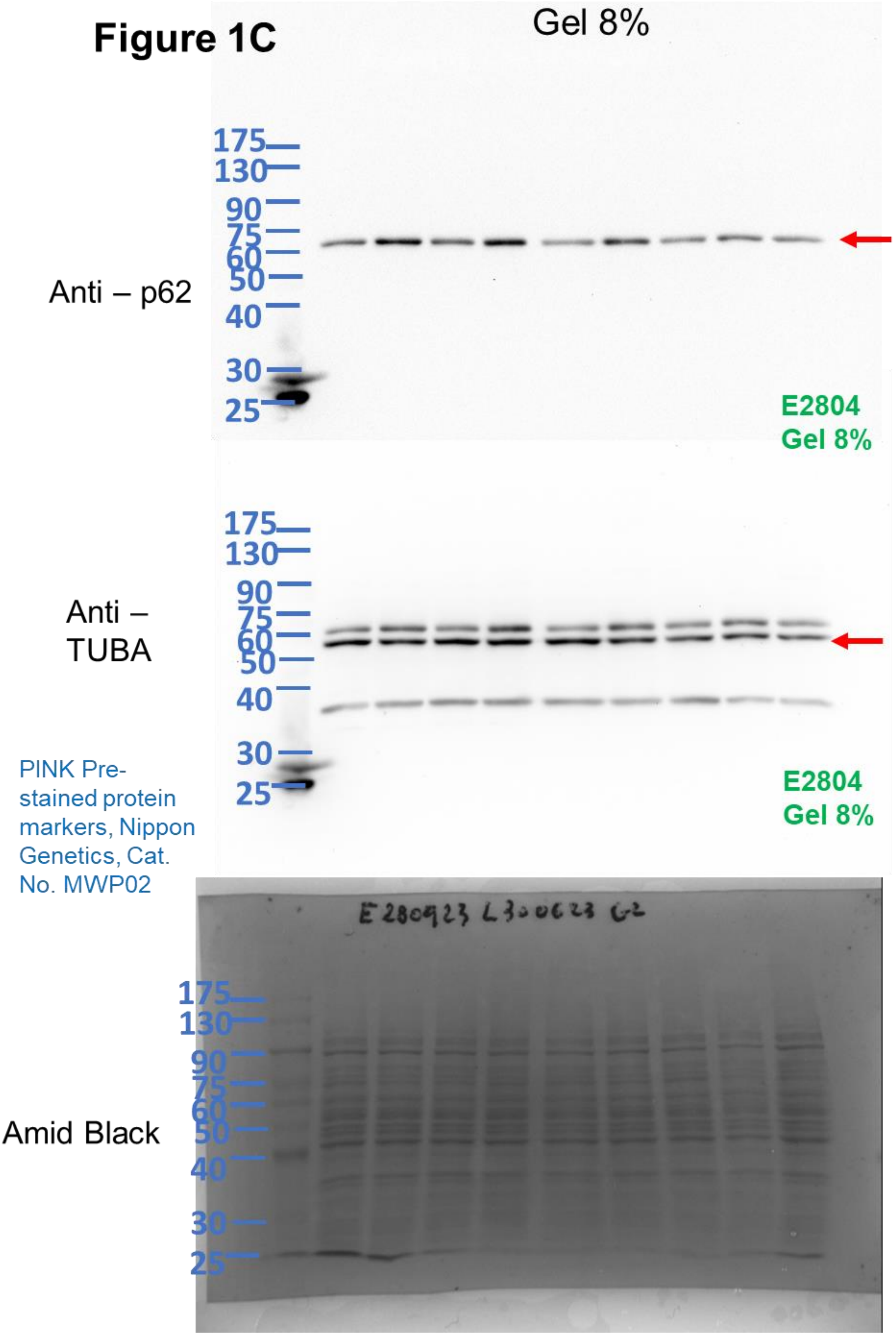
Gel 8%
Anti – p62
175
130
90
75
60
50
40
30
25
E2804
Gel 8%
Anti – TUBA
175
130
90
75
60
50
40
30
25
E2804
Gel 8%
PINK Pre-stained protein markers, Nippon Genetics, Cat. No. MWP02
Amid Black
175
130
90
75
60
50
40
30
25

Figure 1E

gel 15%

Anti - LC3

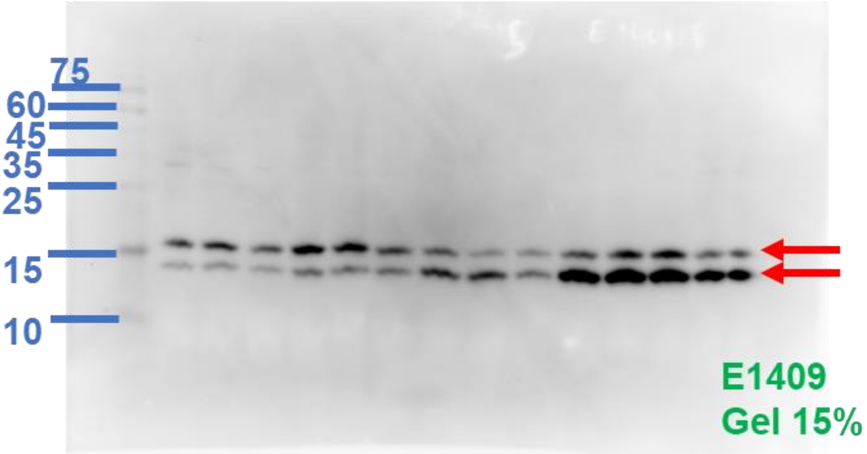

Figure 1E
Figure S2F

Anti - GAPDH

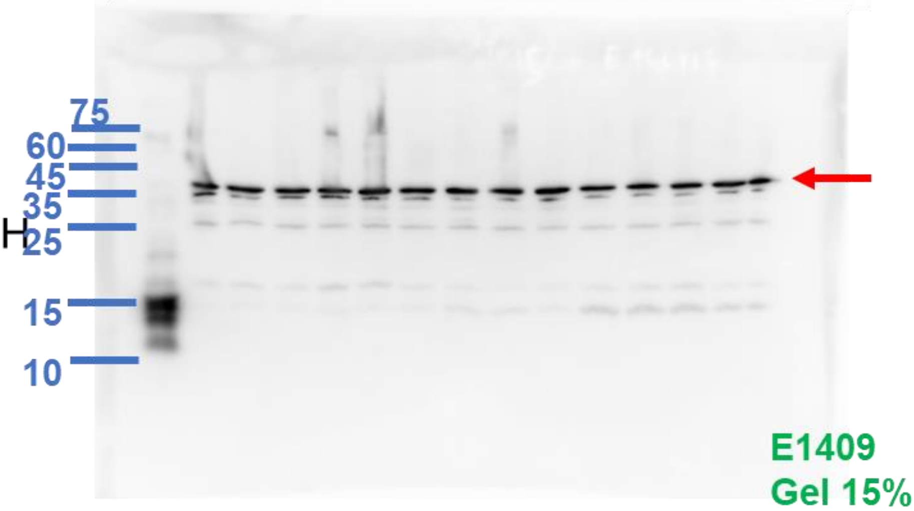

Figure S2F

Anti – NQO2

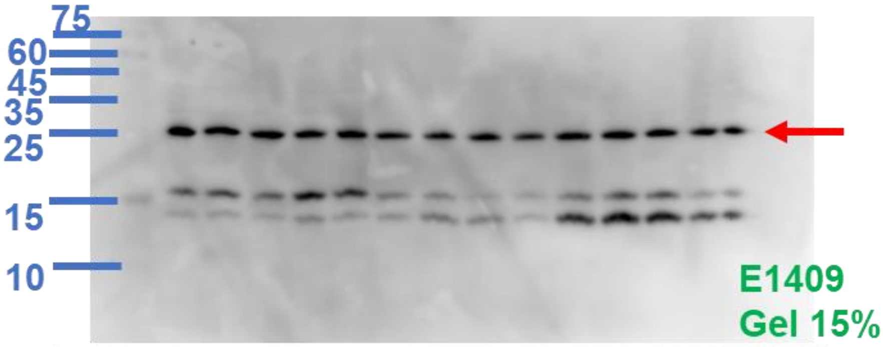

PINK Pre-stained protein markers, Nippon Genetics, Cat. No. MWP02

Amid Black

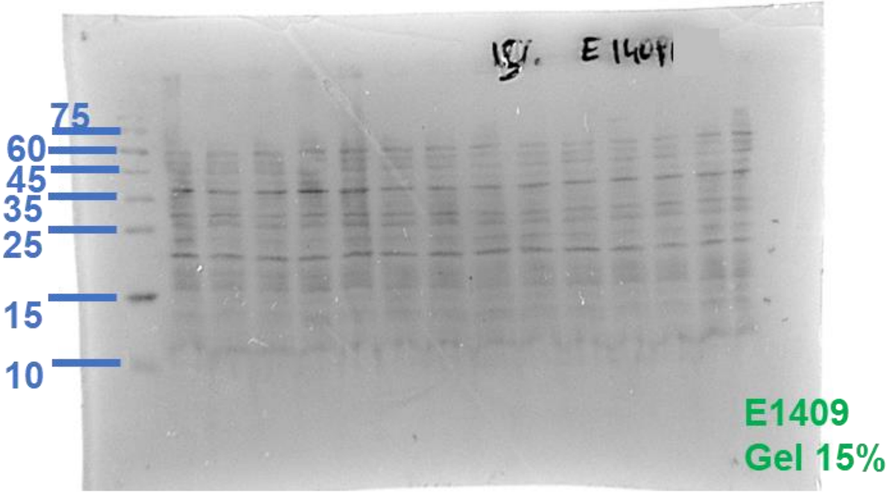

Figure 1E

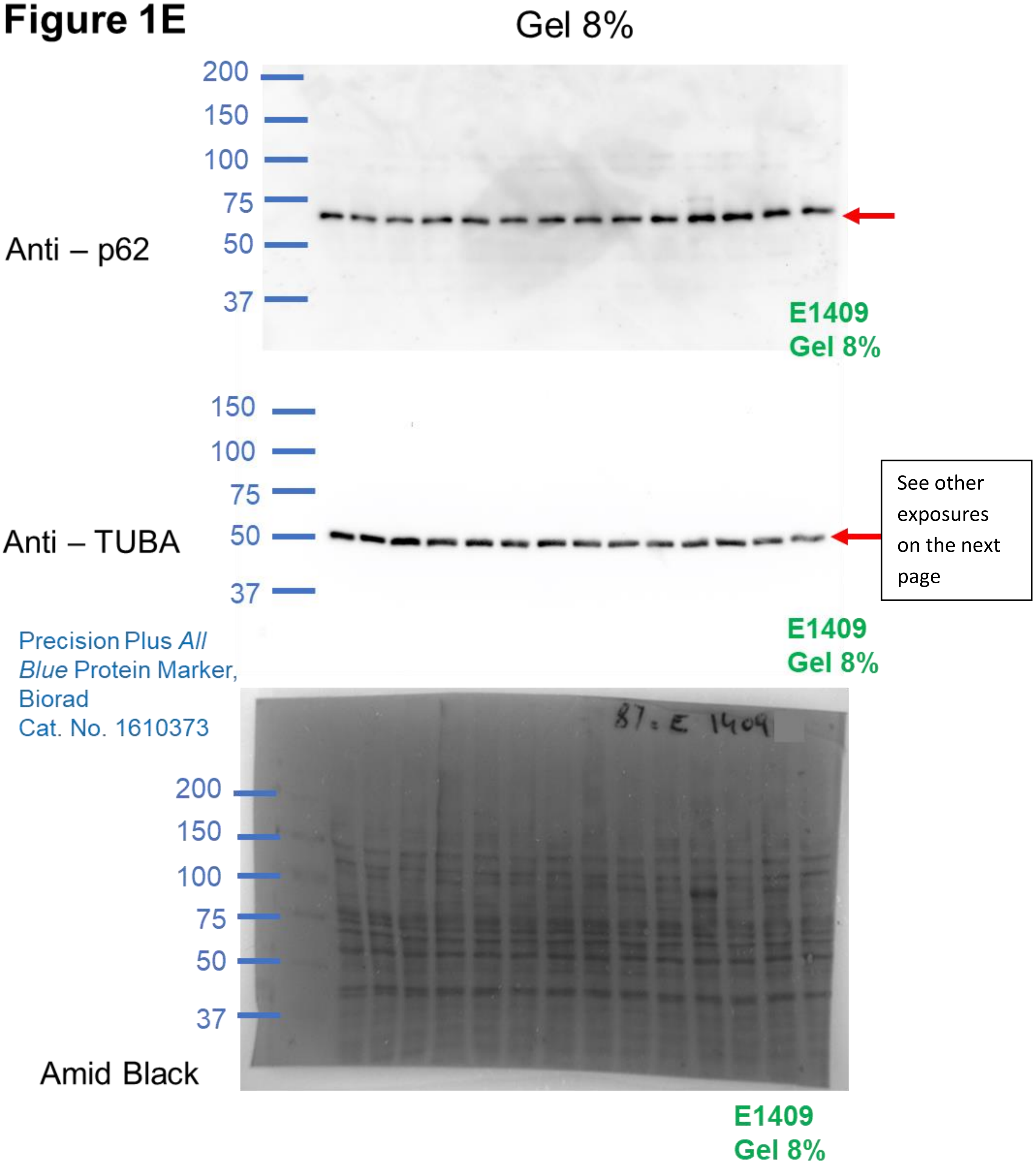
Gel 8%
200
150
100
75
50
37
Anti – p62
E1409
Gel 8%
150
100
75
50
37
Anti – TUBA
See other exposures on the next page
E1409
Gel 8%
Precision Plus *All Blue* Protein Marker, Biorad
Cat. No. 1610373
87: E 1409
200
150
100
75
50
37
Amid Black
E1409
Gel 8%

Figure 1E

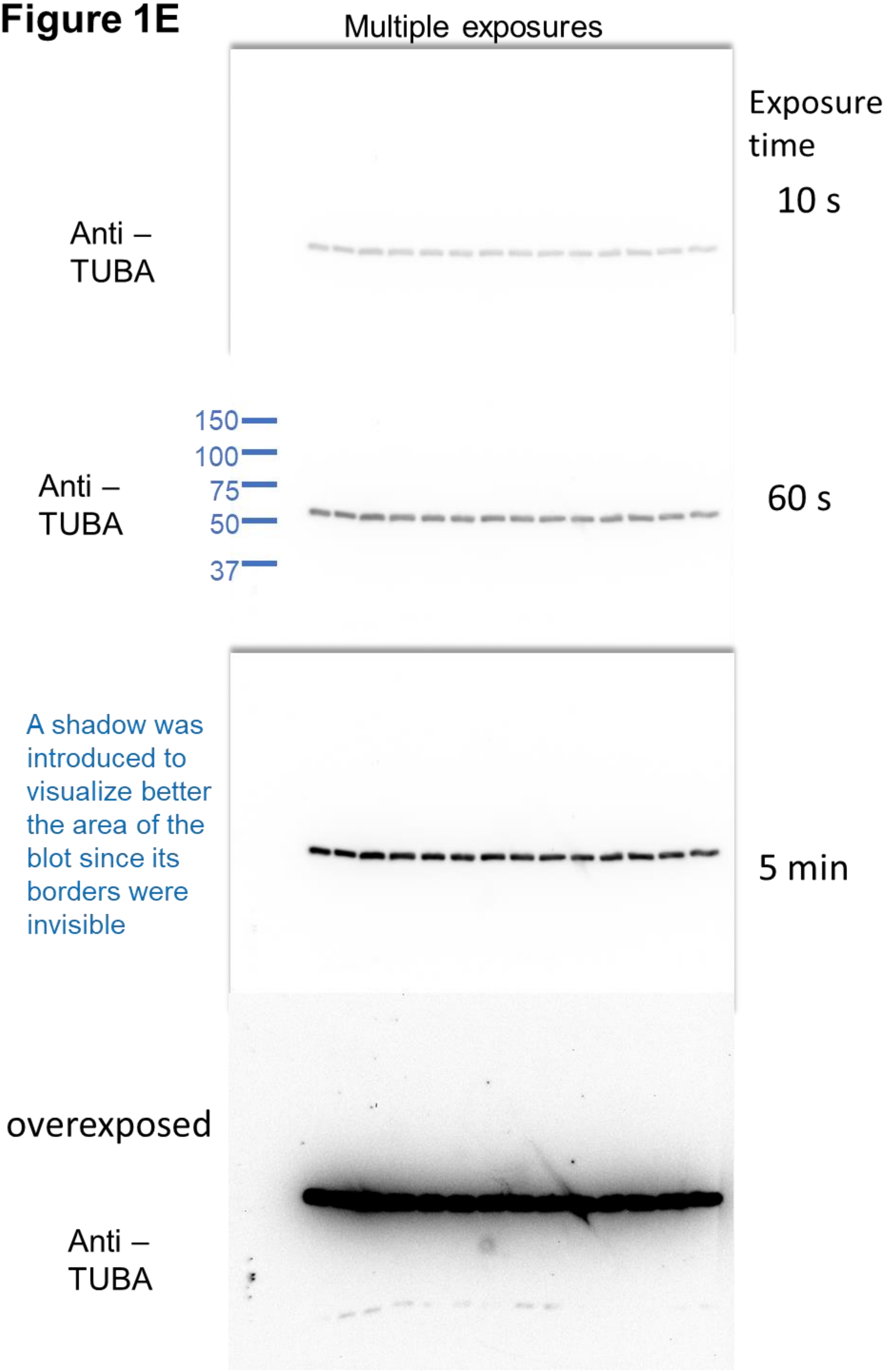
Multiple exposures
Exposure time
10 s
Anti – TUBA
150
100
75
50
37
Anti – TUBA
60 s
A shadow was introduced to visualize better the area of the blot since its borders were invisible
5 min
overexposed
Anti – TUBA

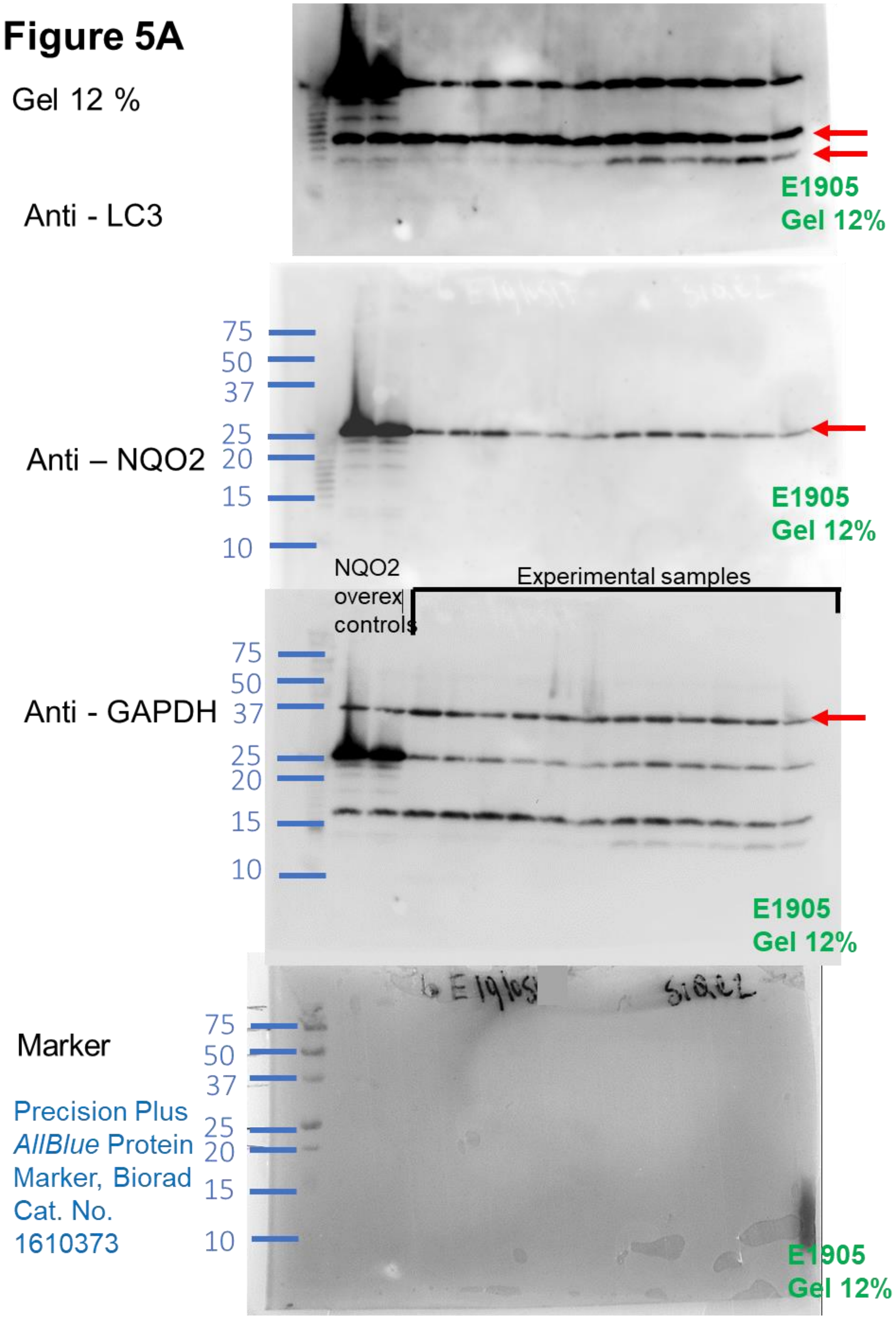
Figure 5A
Gel 12 %
Anti - LC3
E1905
Gel 12%
Anti – NQO2
75
50
37
25
20
15
10
E1905
Gel 12%
NQO2
overex
controls
Experimental samples
Anti - GAPDH
75
50
37
25
20
15
10
E1905
Gel 12%
Marker
Precision Plus
AllBlue Protein
Marker, Biorad
Cat. No.
1610373
75
50
37
25
20
15
10
E1905
Gel 12%

Figure 5C

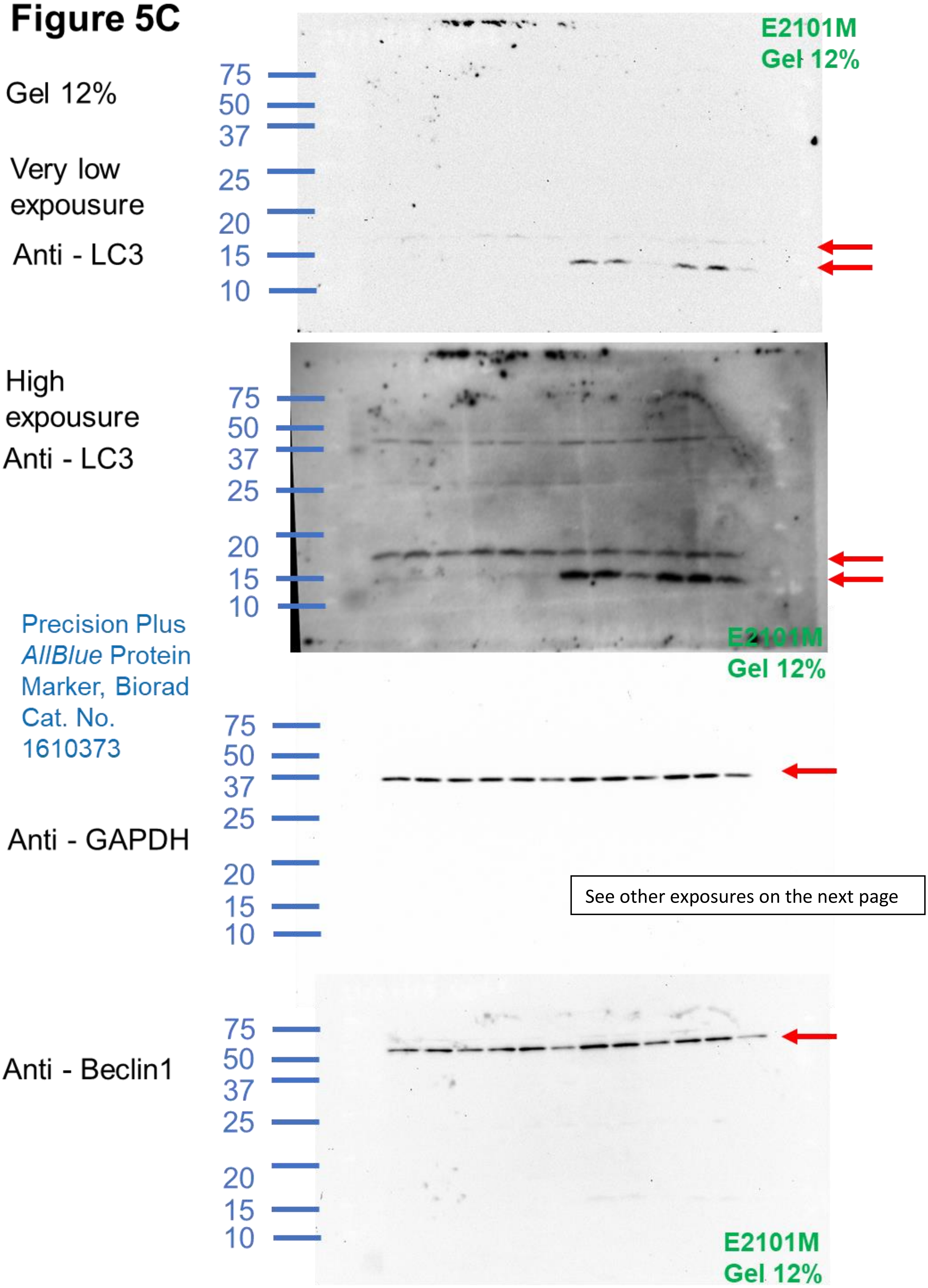

See other exposures on the next page

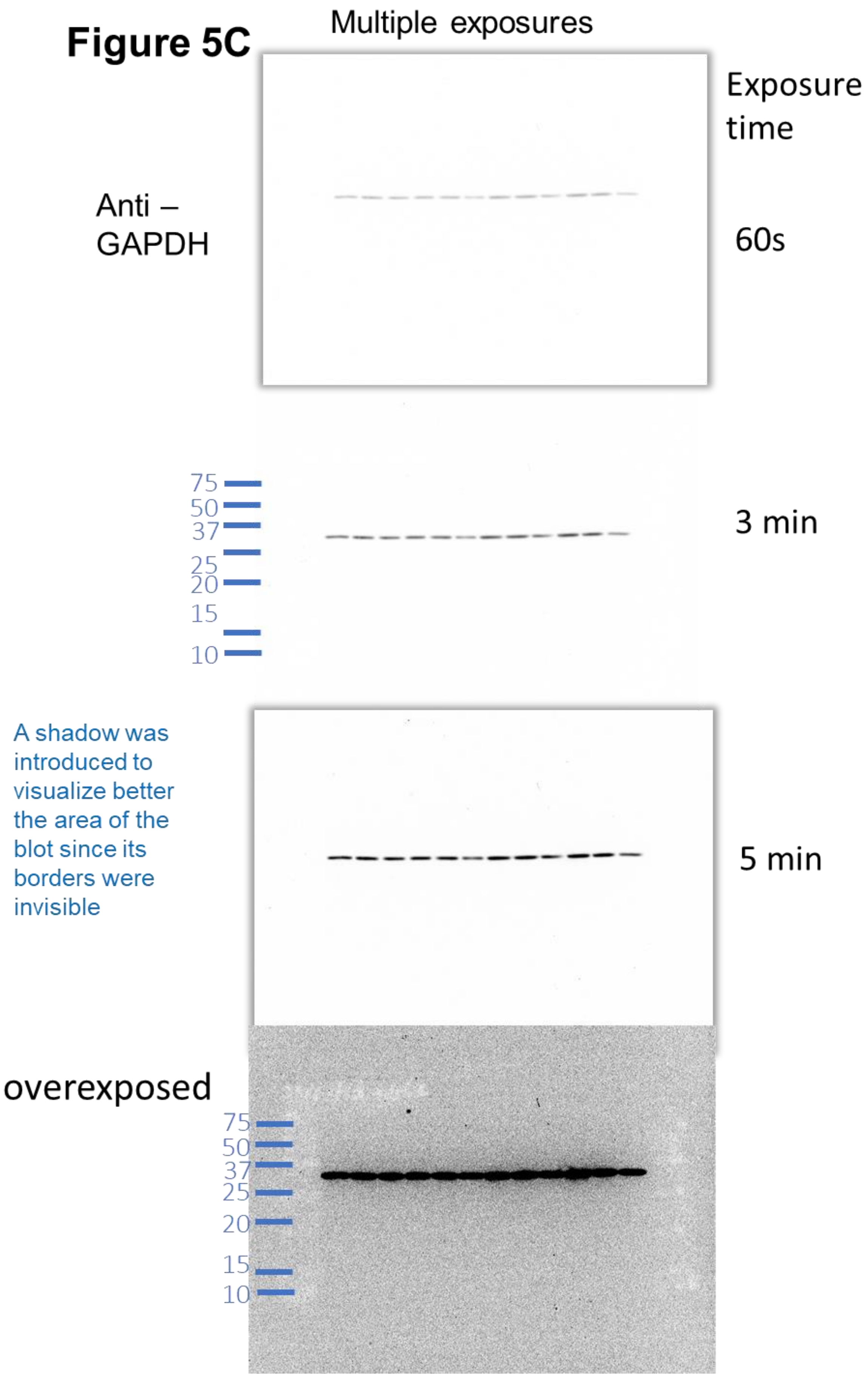
Figure 5C
Multiple exposures
Exposure time
Anti – GAPDH
60s
75
50
37
25
20
15
10
3 min
A shadow was introduced to visualize better the area of the blot since its borders were invisible
5 min
overexposed
75
50
37
25
20
15
10

Figure 5E

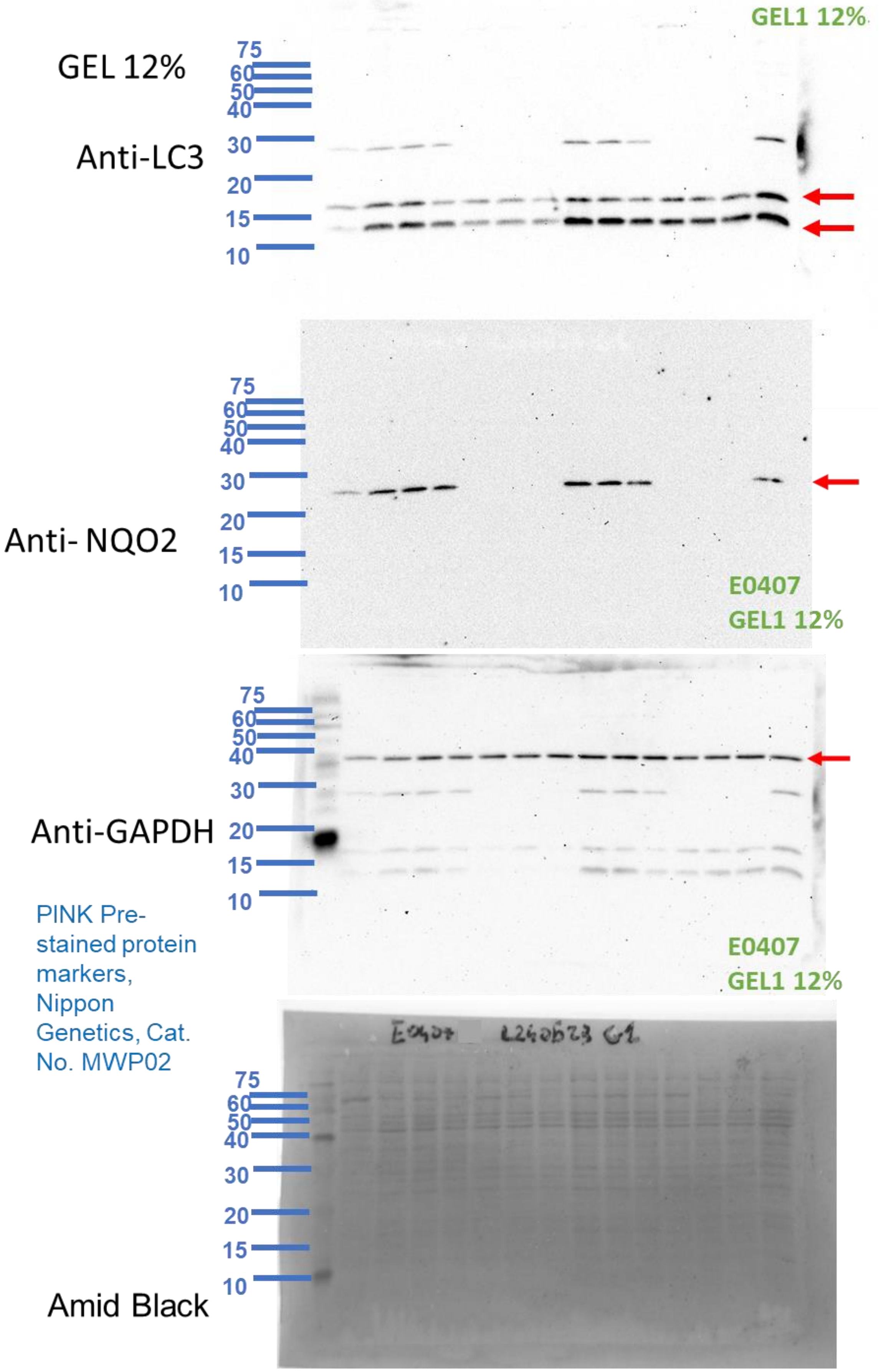
E0407
GEL1 12%
GEL 12%
Anti-LC3
75
60
50
40
30
20
15
10
Anti- NQO2
Anti-GAPDH
PINK Pre-stained protein markers, Nippon Genetics, Cat. No. MWP02
Amid Black

Figure 5E

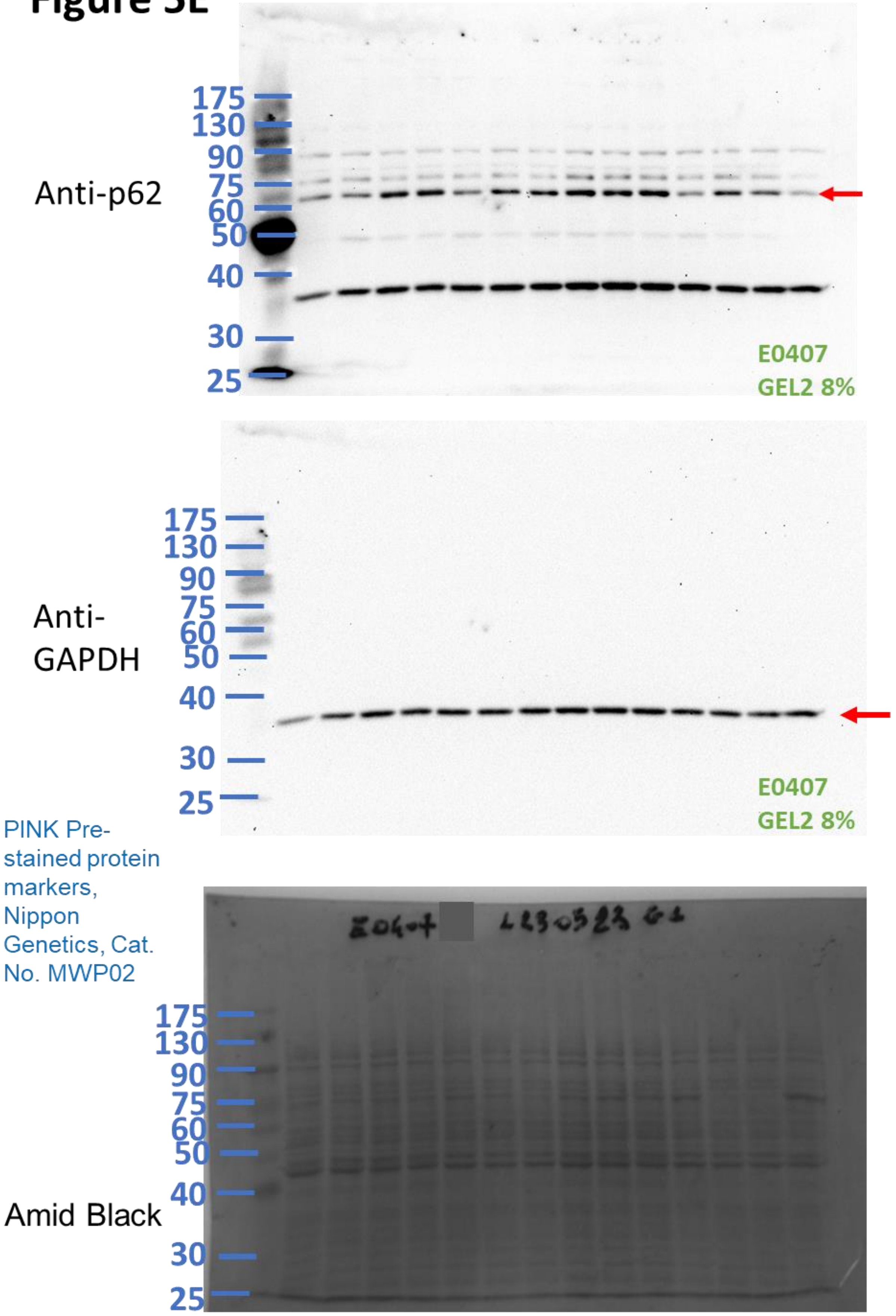
Anti-p62
175
130
90
75
60
50
40
30
25
E0407
GEL2 8%
Anti-GAPDH
175
130
90
75
60
50
40
30
25
E0407
GEL2 8%
PINK Pre-stained protein markers, Nippon Genetics, Cat. No. MWP02
Amid Black
175
130
90
75
60
50
40
30
25

Figure 6E

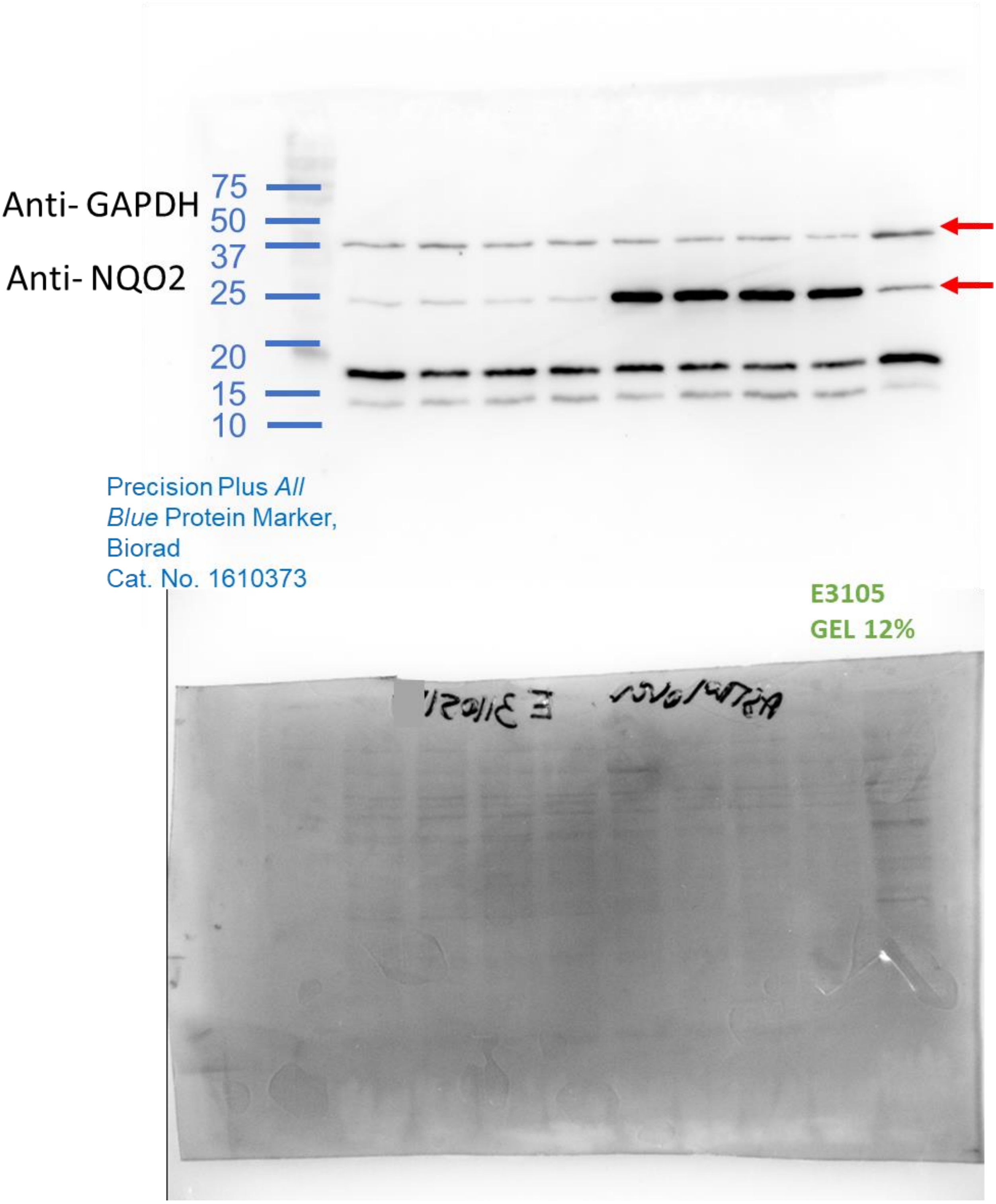
Anti- GAPDH
Anti- NQO2
75
50
37
25
20
15
10
Precision Plus *All Blue* Protein Marker, Biorad
Cat. No. 1610373
E3105
GEL 12%

Figure S1A

Gel 15%

Anti - LC3

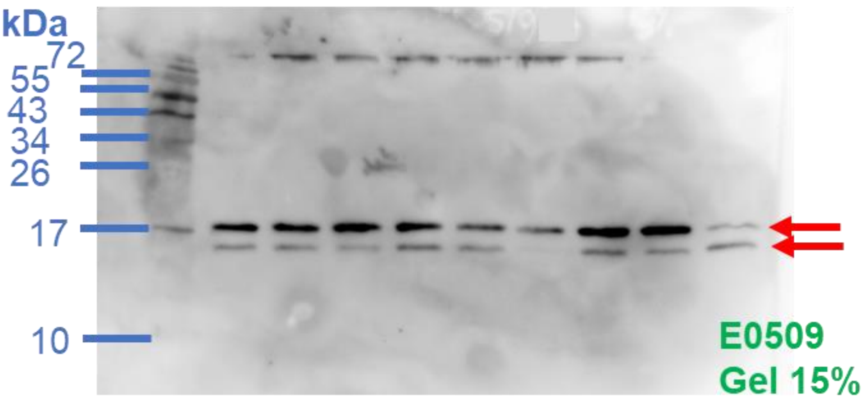

Figure S1A

Figure S2D

Anti - GAPDH

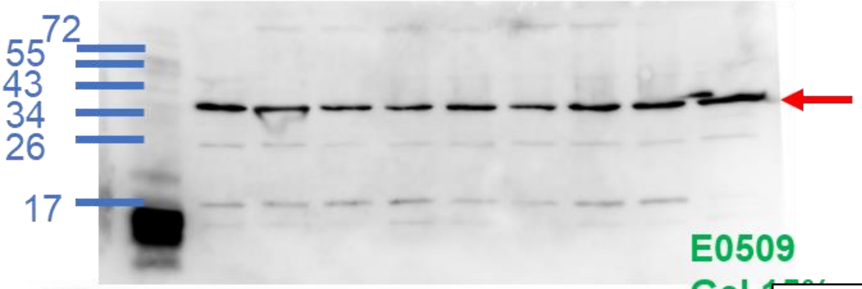

See other exposures on the next page

Figure S2D

Anti – NQO2

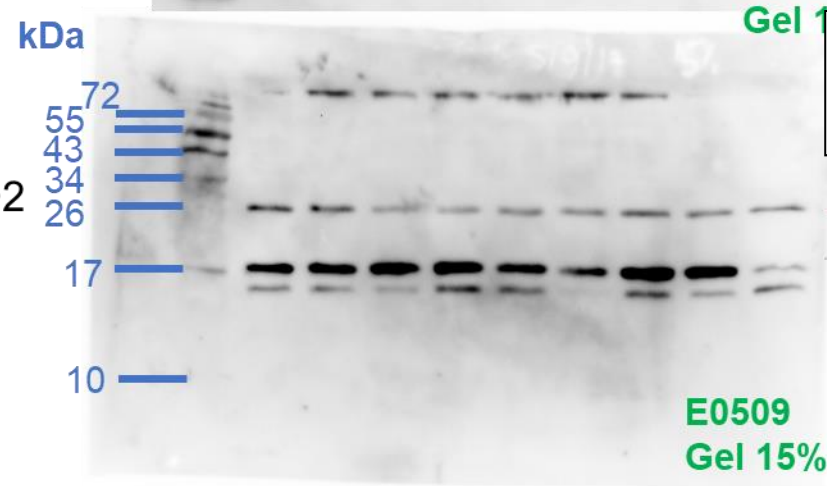

Figure S1A

Figure S2D

Fisher BioReagents EZ-Run Prestained *Rec* Protein Marker Cat. BP3603-1

Amid Black

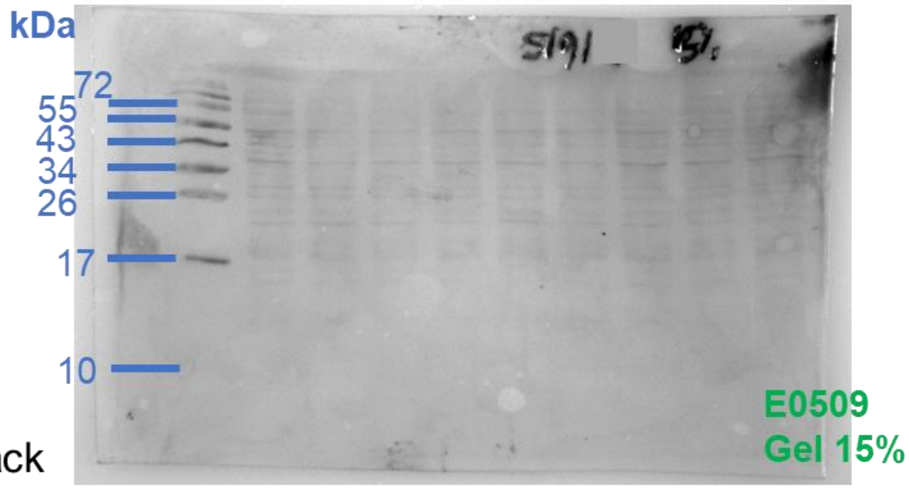

Figure S1A

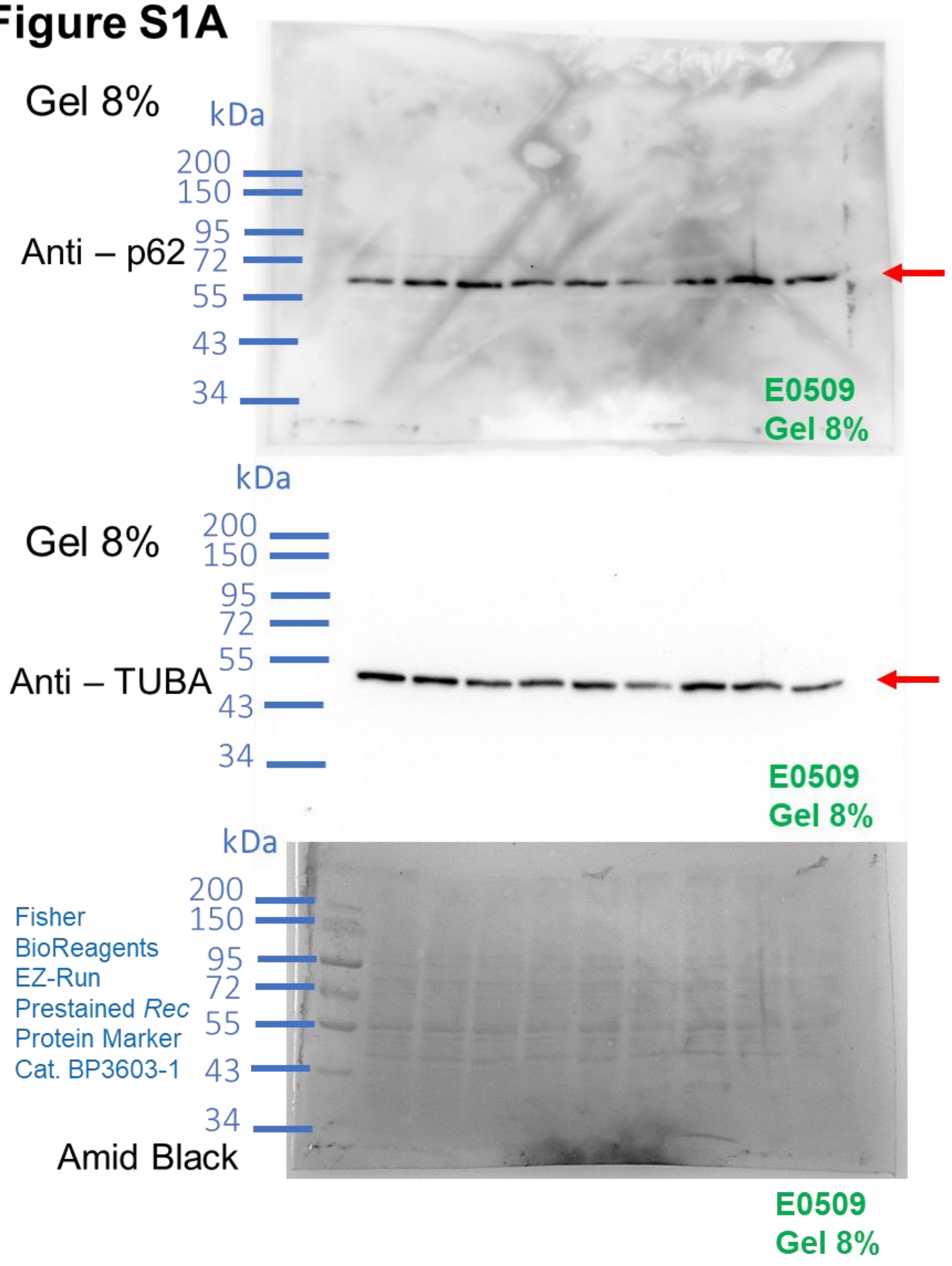
Gel 8%
kDa
200
150
95
72
55
43
34
Anti – p62
E0509
Gel 8%
kDa
Gel 8%
200
150
95
72
55
43
34
Anti – TUBA
E0509
Gel 8%
kDa
200
150
95
72
55
43
34
Fisher BioReagents EZ-Run Prestained *Rec* Protein Marker Cat. BP3603-1
Amid Black
E0509
Gel 8%

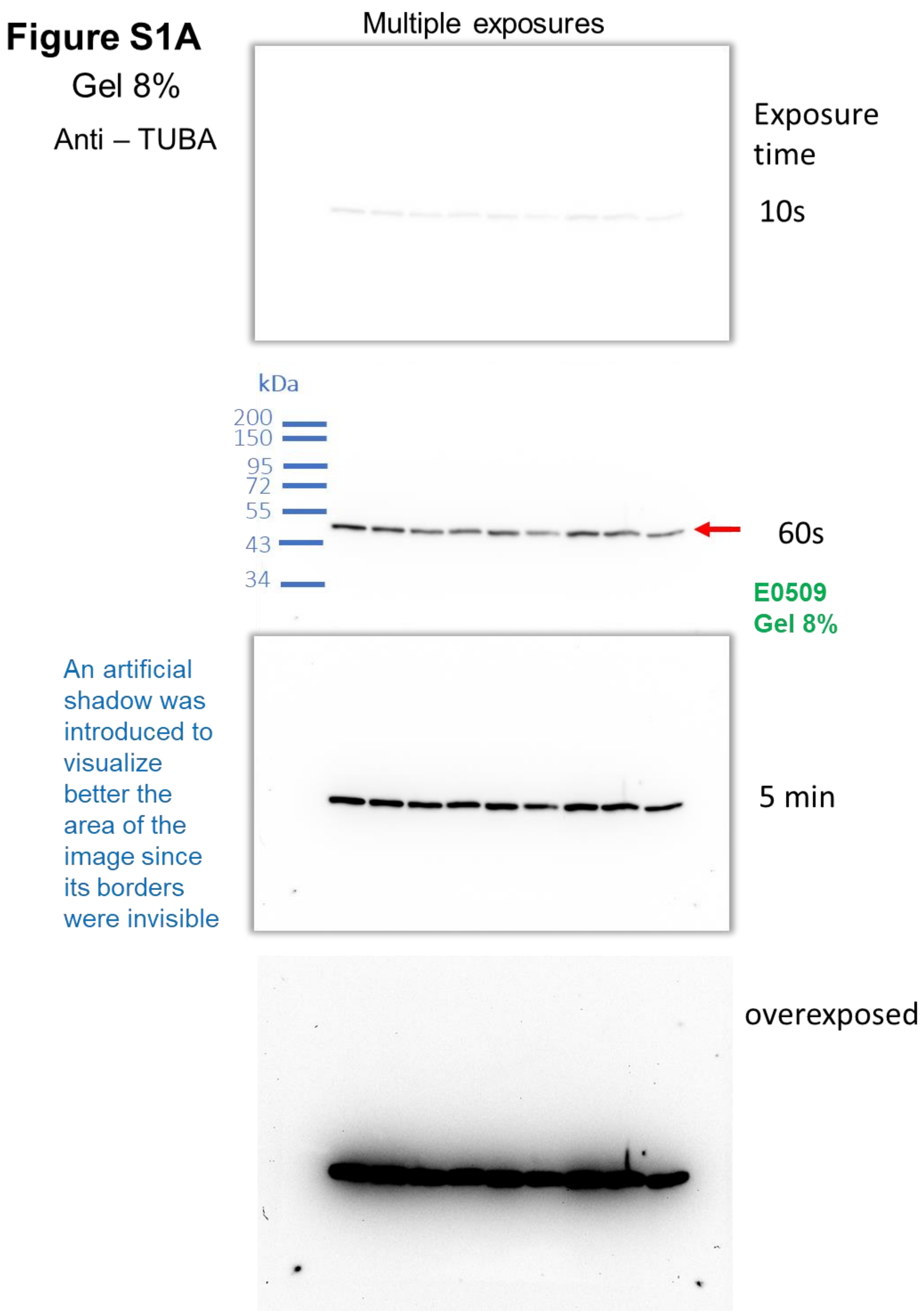
Figure S1A
Gel 8%
Anti – TUBA
Multiple exposures
Exposure time
10s
kDa
200
150
95
72
55
43
34
60s
E0509
Gel 8%
An artificial shadow was introduced to visualize better the area of the image since its borders were invisible
5 min
overexposed

Figure S2A whole blots are presented together with the Fig. 1A

Figure S2C

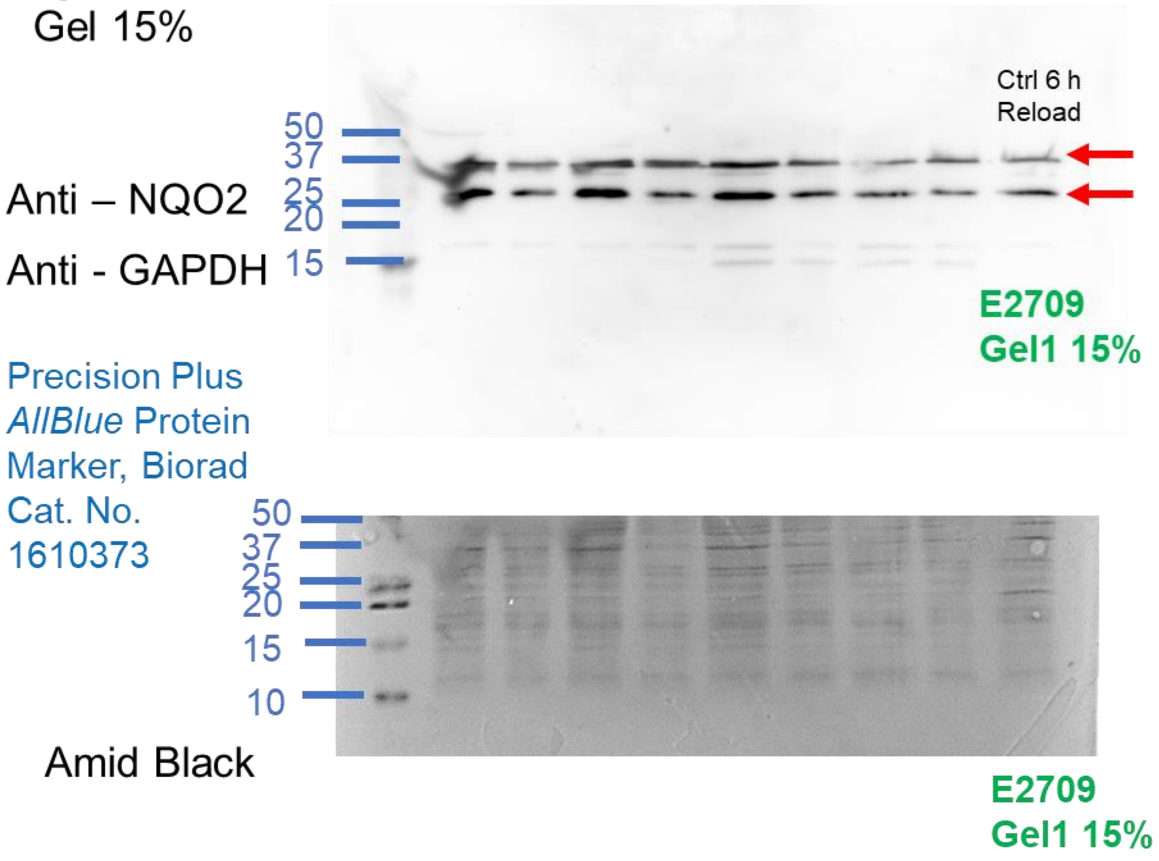

Figure S2D whole blots are presented together with the Fig. S1A Gel 15%

Figure S2E see next page

Figure S2F whole blots are presented together with the Fig. 1E Gel 15%

Figure S2E

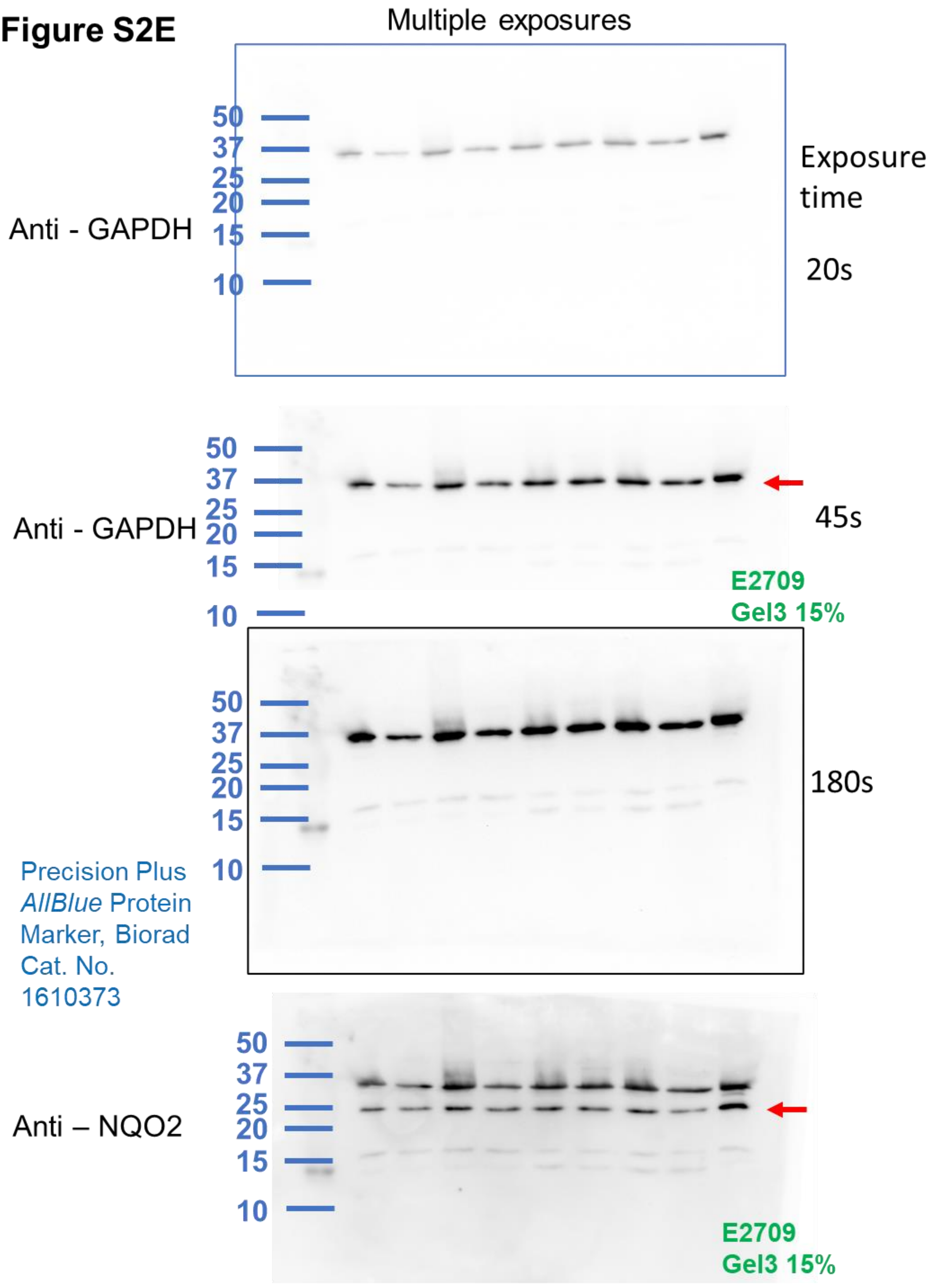
Multiple exposures
Anti - GAPDH
50
37
25
20
15
10
Exposure time
20s
Anti - GAPDH
50
37
25
20
15
10
45s
E2709
Gel3 15%
50
37
25
20
15
10
180s
Precision Plus AllBlue Protein Marker, Biorad Cat. No. 1610373
Anti – NQO2
50
37
25
20
15
10
E2709
Gel3 15%
